# IFI16 Restricts HSV-1 Replication by Accumulating on the HSV-1 Genome, Repressing HSV-1 Gene Expression, and Directly or Indirectly Modulating Histone Modifications

**DOI:** 10.1371/journal.ppat.1004503

**Published:** 2014-11-06

**Authors:** Karen E. Johnson, Virginie Bottero, Stephanie Flaherty, Sujoy Dutta, Vivek Vikram Singh, Bala Chandran

**Affiliations:** H.M. Bligh Cancer Research Laboratories, Department of Microbiology and Immunology, Chicago Medical School, Rosalind Franklin University of Medicine and Science, North Chicago, Illinois, United States of America; University of Glasgow, United Kingdom

## Abstract

Interferon-γ inducible factor 16 (IFI16) is a multifunctional nuclear protein involved in transcriptional regulation, induction of interferon-β (IFN-β), and activation of the inflammasome response. It interacts with the sugar-phosphate backbone of dsDNA and modulates viral and cellular transcription through largely undetermined mechanisms. IFI16 is a restriction factor for human cytomegalovirus (HCMV) and herpes simplex virus (HSV-1), though the mechanisms of HSV-1 restriction are not yet understood. Here, we show that IFI16 has a profound effect on HSV-1 replication in human foreskin fibroblasts, osteosarcoma cells, and breast epithelial cancer cells. IFI16 knockdown increased HSV-1 yield 6-fold and IFI16 overexpression reduced viral yield by over 5-fold. Importantly, HSV-1 gene expression, including the immediate early proteins, ICP0 and ICP4, the early proteins, ICP8 and TK, and the late proteins gB and Us11, was reduced in the presence of IFI16. Depletion of the inflammasome adaptor protein, ASC, or the IFN-inducing transcription factor, IRF-3, did not affect viral yield. ChIP studies demonstrated the presence of IFI16 bound to HSV-1 promoters in osteosarcoma (U2OS) cells and fibroblasts. Using CRISPR gene editing technology, we generated U2OS cells with permanent deletion of IFI16 protein expression. ChIP analysis of these cells and wild-type (wt) U2OS demonstrated increased association of RNA polymerase II, TATA binding protein (TBP) and Oct1 transcription factors with viral promoters in the absence of IFI16 at different times post infection. Although IFI16 did not alter the total histone occupancy at viral or cellular promoters, its absence promoted markers of active chromatin and decreased those of repressive chromatin with viral and cellular gene promoters. Collectively, these studies for the first time demonstrate that IFI16 prevents association of important transcriptional activators with wt HSV-1 promoters and suggest potential mechanisms of IFI16 restriction of wt HSV-1 replication and a direct or indirect role for IFI16 in histone modification.

## Author Summary

HSV-1, a ubiquitous human pathogen that establishes a life-long infection, has evolved several mechanisms to evade host immune detection and responses. However, it is still subject to regulation by cellular factors. Recently, a host nuclear protein, IFI16, was shown to be involved in the innate defense response to HSV-1 infection. Here, we provide the first evidence that IFI16 inhibits wild-type HSV-1 replication by repressing viral gene expression independent of its roles in the immune response. We show that IFI16 binds the HSV-1 genome at the transcription start sites of several HSV-1 genes. Using a permanently IFI16-negative cell line that we generated, we demonstrate that IFI16 reduces the association of important transcription factors. IFI16 also promotes global histone modifications by increasing the markers of repressive chromatin and decreasing the markers for activating chromatin on viral and cellular genes. These insights into the role of IFI16 in HSV-1 biology suggest that stabilization of IFI16 is an attractive avenue for antiviral drug development.

## Introduction

Herpes simplex virus type I (HSV-1) is a ubiquitous and highly contagious virus that establishes a life-long infection in host organisms. It typically enters the host through mucosal epithelia and causes a lytic, productive infection in many cell types, including fibroblast, epithelial, and endothelial cells, during which more than 80 gene products are produced from the nuclear viral genome. After primary infection, HSV-1 spreads to neuronal cells in the trigeminal ganglia where it establishes latent infection, during which only the Latency Associated Transcript (LAT) is produced. Periodically, HSV-1 is reactivated from latency and causes recurrent lytic infection at the site of primary infection [1]. HSV-1 typically causes oral lesions but can cause much more severe pathologies, including blindness and fatal encephalitis, due to its infection of corneal cells and the central nervous system [2]–[4].

During lytic infection, HSV-1 genes are transcribed by cellular RNA polymerase II (RNA pol II), assisted by cellular transcription factors, including TATA-binding protein (TBP), in a highly regulated temporal cascade. Transcription from the immediate early (IE) gene promoters of HSV-1 begins as soon as the viral genome enters the nucleus and is initiated by the virion tegument-associated protein, VP16, in conjunction with the cellular transcription factors, Oct1 and HCF. Most IE genes regulate viral and cellular gene expression. The next temporal class of HSV-1 genes, early (E) genes, is expressed around 2–8 hours post-infection (h p.i.) and is largely involved in DNA replication. Expression of these genes is dependent on the viral IE regulatory proteins, ICP0 and ICP4, and cellular RNA pol II, TBP, and other transcription factors. The final broad category of HSV-1 gene expression, late (L) genes, is further categorized into leaky late (DNA replication-independent) and true late (DNA replication-dependent) genes. Late genes encode predominantly structural proteins and their expression is also dependent on viral ICP4, and host RNA pol II, and TBP proteins [1], [5], [6]

Because of its lifelong infection of human hosts, HSV-1 has necessarily evolved a complex set of interactions with host cell factors to modulate host and viral gene expression and to evade immune detection and responses. In addition to the gene regulation outlined above, HSV-1 inhibits cellular gene expression to better co-opt the gene expression machinery for itself. The virion host shut off (vhs) protein inhibits protein synthesis by causing degradation of host and cellular mRNAs [7], [8] and, conversely, enhancing expression of late viral mRNAs [9]. Similarly, ICP34.5, a late protein, dephosphorylates host eIF2α and inhibits protein synthesis [10].

The virion-associated HSV-1 genomic DNA associates with the histones and nucleosome proteins, leading into its chromatinization and the epigenetic control of viral genes [11]–[16]. Histones can be shifted along DNA or modified, leading to the condensation (heterochromatin) or relaxation (euchromatin) of chromatin, resulting in the suppression or activation of gene expression, respectively. Many of these functional histone modifications occur on histone H3, including markers for heterochromatin, including trimethylated H3 lysine 9 (H3K9me3) or lysine 27 (H3K27me3), and markers for euchromatin, such as trimethylated lysine 4 (H3K4me3) or acetylation of lysines K9, K14, or K27 [17]. Several viral gene products, including ICP0, VP16, and LAT have been implicated in viral chromatin remodeling during HSV-1 infection [12]–[14], [16], [18].

Along with these basic gene expression and replication interactions with host factors, HSV-1 regulates the host immune response by several mechanisms. ICP0, a transcriptional regulator and E3 ubiquitin ligase, induces the degradation of antiviral factors [19]–[21] and inhibits type 1 IFN expression [20], [22], [23]. HSV-1 has also evolved mechanisms to inhibit IFN signaling [24]–[27]. Our recent studies have shown that HSV-1 activates and then represses the inflammasome response [19].

IFN-γ inducible protein 16 (IFI16) is a DNA binding protein [28], [29] that was first described as an IFN-inducible protein involved in the differentiation of human myeloid cells [30], [31]. It is a transcriptional modulator [32] through mechanisms that are not yet fully defined. IFI16 interacts with p53 [33] and regulates p53 target gene expression [34]. Interestingly, both loss and gain of IFI16 induces p53 checkpoints; overexpression leads to the apoptotic p53 checkpoint and loss of IFI16 leads to cell cycle arrest [35], [36]. IFI16 is acetylated by the histone acetyltransferase, p300 [37], and may play a role in regulating gene expression by modulating chromatinization [38].

Several immunomodulatory roles have been described for IFI16. It recognizes nuclear herpesviral genomes, including those of HSV-1, Kaposi's sarcoma-associated herpesvirus (KSHV), and Epstein-Barr virus (EBV), and responds to human immunodeficiency virus (HIV) infection, leading to association with apoptosis-associated speck-like protein containing a caspase activation and recruitment (CARD) domain (ASC) through its CARD domain to induce inflammasome activity, resulting in the maturation of caspase-1 and IL-1β [19], [39]–[42]. IFI16 is also necessary for HSV-1-, human cytomegalovirus (HCMV)-, and Vaccinia-virus-induced STING-mediated IFNβ expression [20], [28], [43]. Interestingly, HSV-1 induces the specific degradation of IFI16 at late times post-infection (after 4 h), dependent, at least in part, on ICP0 [19], [20], [44].

Recently, IFI16 has been described as a restriction factor for herpesviral lytic replication [38], [44], [45]. It restricts HCMV replication by displacing transcription factors on E and L but not IE gene promoters [45] and restricts HSV-1 replication [38], [44], [45], particularly replication of ICP0-deficient HSV-1 [38], [44]. IFI16 promotes association of repressive histone modifications with the ICP4, ICP27, and ICP8 HSV-1 promoters during infection with this mutant virus that lacks ICP0 but had no apparent effect on histone modifications associated with viral promoters during infection during infection with a rescue virus [38]. In addition, viral gene expression was repressed, somewhat, during infection with this ICP0-null virus in IFI16-positive cells compared with that in IFI16-depleted cells, but viral gene expression of ICP0-competent rescue HSV-1 was not affected [38]. Though this study suggested that IFI16 recognizes and regulates unchromatinized DNA [38], observations such as the recognition of chromatinized KSHV and EBV DNA by IFI16 during latency, persistence of latent KSHV and EBV gene expression in the presence of IFI16 and the IFI16-inflammasome [39], [40], [42] and the repressive effect of IFI16 on wild-type (wt) HSV-1 replication observed previously [45] or in this study suggest that the mechanisms of IFI16 restriction of HSV-1 are complex.

The study showing repression of HCMV replication by IFI16 demonstrated IFI16-mediated repression of HSV-1 replication but did not pursue mechanisms, thereof [45]. There are multiple differences in cell tropism, replication kinetics, and immune evasion strategies evolved by HSV-1 and HCMV [1], [37]. Notably, HSV-1 causes the specific degradation of IFI16 while HCMV does not [19], [20], [37], [44]. Therefore, we speculated that the mechanisms of HSV-1 restriction by IFI16 may be distinct from those of HCMV restriction.

Here, we show that IFI16 restricts wt HSV-1 replication and gene expression in multiple cell types. It binds to HSV-1 transcription start sites (TSS) of all temporal classes of HSV-1 gene expression, and prevents association of transcription factors, including RNA pol II, TBP, and Oct1 with viral promoters but not cellular promoters. We also show for the first time that IFI16 induces increased euchromatin markers and decreased heterochromatin markers associated with both wt HSV-1 and cellular DNA. These data suggest that IFI16 plays a multi-level role in the modulation of HSV-1 gene repression and that development of drugs to stabilize the function of IFI16 may potentially lead into an effective anti-HSV-1 treatment.

## Results

### IFI16 restricts HSV-1 replication and gene expression in human foreskin fibroblast (HFF) cells

**Figure 1:**
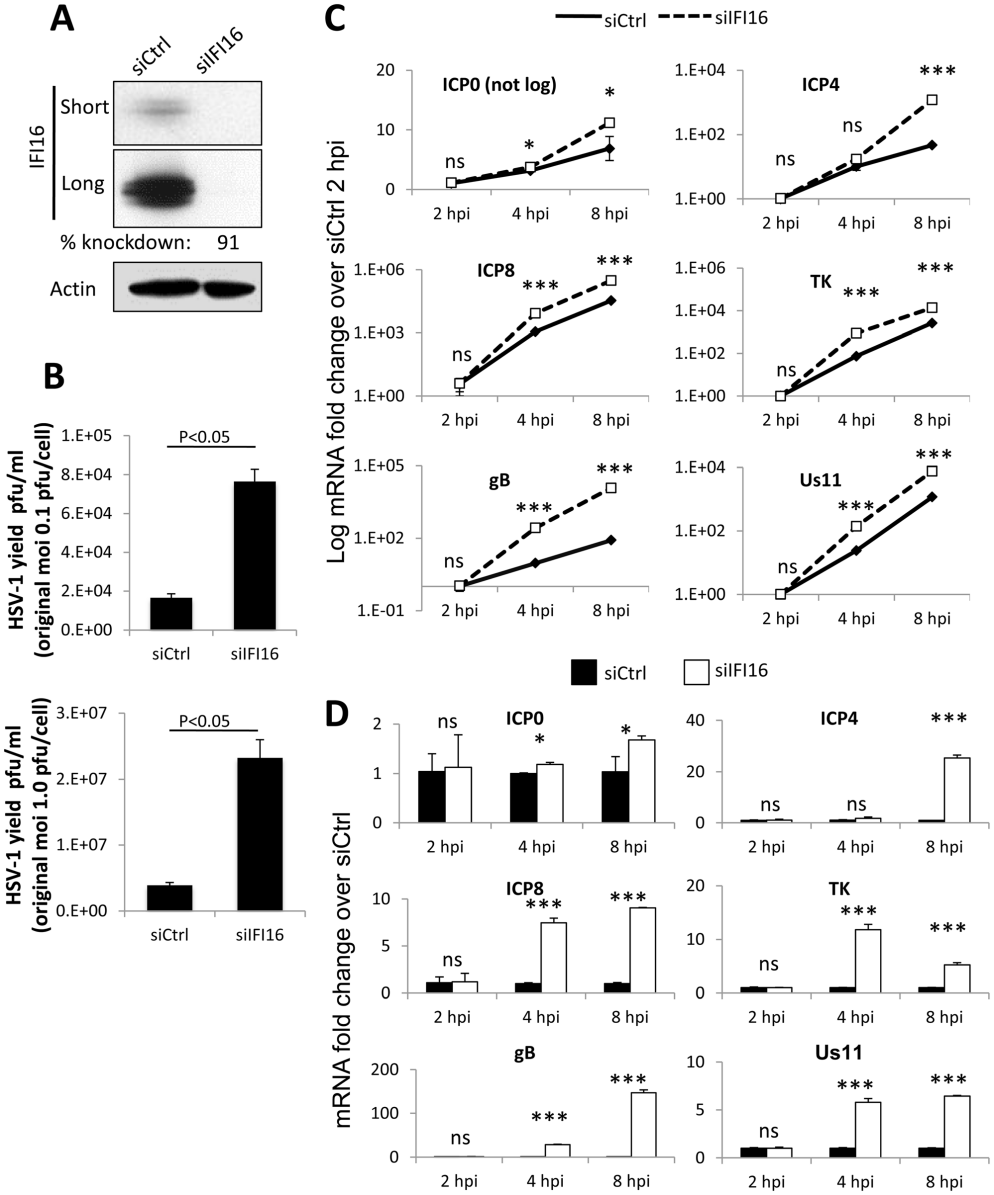
Effect of IFI16 depletion on HSV-1 gene expression, replication and viral yield. HFF cells were microporated with siCtrl or siIFI16 RNA. (A) Western blot analysis of IFI16 knockdown at 48 h post microporation. Shown are short and long exposures of the same blot. Actin is used as a loading control. (B) Viral yield at 24 h p.i. from cells originally infected at an moi of 0.1 pfu/cell (top) or 1 pfu/cell (bottom). (C and D) Representative HSV-1 gene expression from cells infected at an moi of 1 pfu/cell, normalized to GAPDH cDNA and levels at 2 h p.i. Shown are mRNA fold-change over siCtrl at 2 h p.i. (C) or concurrent siCtrl samples (D), ± standard deviation. Statistics were done using Student's T test (ns: not significant, *p<0.05, **p<0.005, ***p<0.0005).

Gariano et al. showed that IFI16 depletion in wt HSV-1-infected cells significantly increased viral yield [45]. However, other studies showed that depletion of IFI16 increased replication of an ICP0-null virus but had no effect on the ICP0-rescue virus or wt HSV-1 strain 17 [38], [44]. To confirm that IFI16 restricts wt HSV-1 (KOS strain) replication, we depleted IFI16 from HFF cells using microporated siIFI16, which resulted in ∼91% knockdown of IFI16 compared with that of siControl (siCtrl) RNA (Figure 1A). To determine the effect of IFI16 depletion, 48 h after microporation we infected the cells with HSV-1 at a multiplicity of infection (moi) of 0.1 or 1.0 plaque forming units (pfu) per cell. At 24 hours post infection (h p.i.), cell culture supernatant was collected and viral yield was determined by plaque assay. Depletion of IFI16 resulted in a significant 5- or 6-fold increase in viral yield (Figure 1B), after infection at an moi of 0.1 or 1.0 pfu/cell, respectively. This inhibition is consistent with that observed previously for wt HSV-1, but further suggests a different mechanism of HSV-1 repression by IFI16 than that involved in repression of HCMV, which was moi-dependent [45]. Interestingly, this reduction in ICP0-positive HSV-1 yield observed here and previously [45] is inconsistent with other reports, showing IFI16-induced inhibition of an ICP0-null virus but not the ICP0-positive rescue virus or wt strain 17 virus [38], [44].

To determine the effect of IFI16 depletion on HSV-1 gene expression, we infected HFF cells microporated with siCtrl or siIFI16 with HSV-1 at an moi of 1 pfu/cell for 2, 4, or 8 h and determined the relative expression of IE (ICP0 and ICP4), E (ICP8 and TK) and L (gB and Us11) gene mRNA by qRT-PCR and normalized data to GAPDH in each sample and to siCtrl at 2 h p.i. (Figure 1C) or to concurrent siCtrl samples (Figure 1D), using the ddCt method [46]. Expression of each gene increased over the course of infection; however, the increase was significantly further augmented in the absence of IFI16 (Figure 1C). Compared to the expression in siCtrl-transfected cells, IFI16 depletion led to an increase of expression of all gene classes: at 8 h p.i., ICP0 expression increased 1.5-fold, ICP4 expression was increased by about 24-fold, ICP8 increased by 8-fold, TK increased by 12-fold, gB increased 150-fold, and Us11 increased by 12-fold (Figure 1D).

### IFI16 restricts HSV-1 replication and gene expression in human osteosarcoma U2OS cells and breast cancer epithelial MCF7 cells

**Figure 2:**
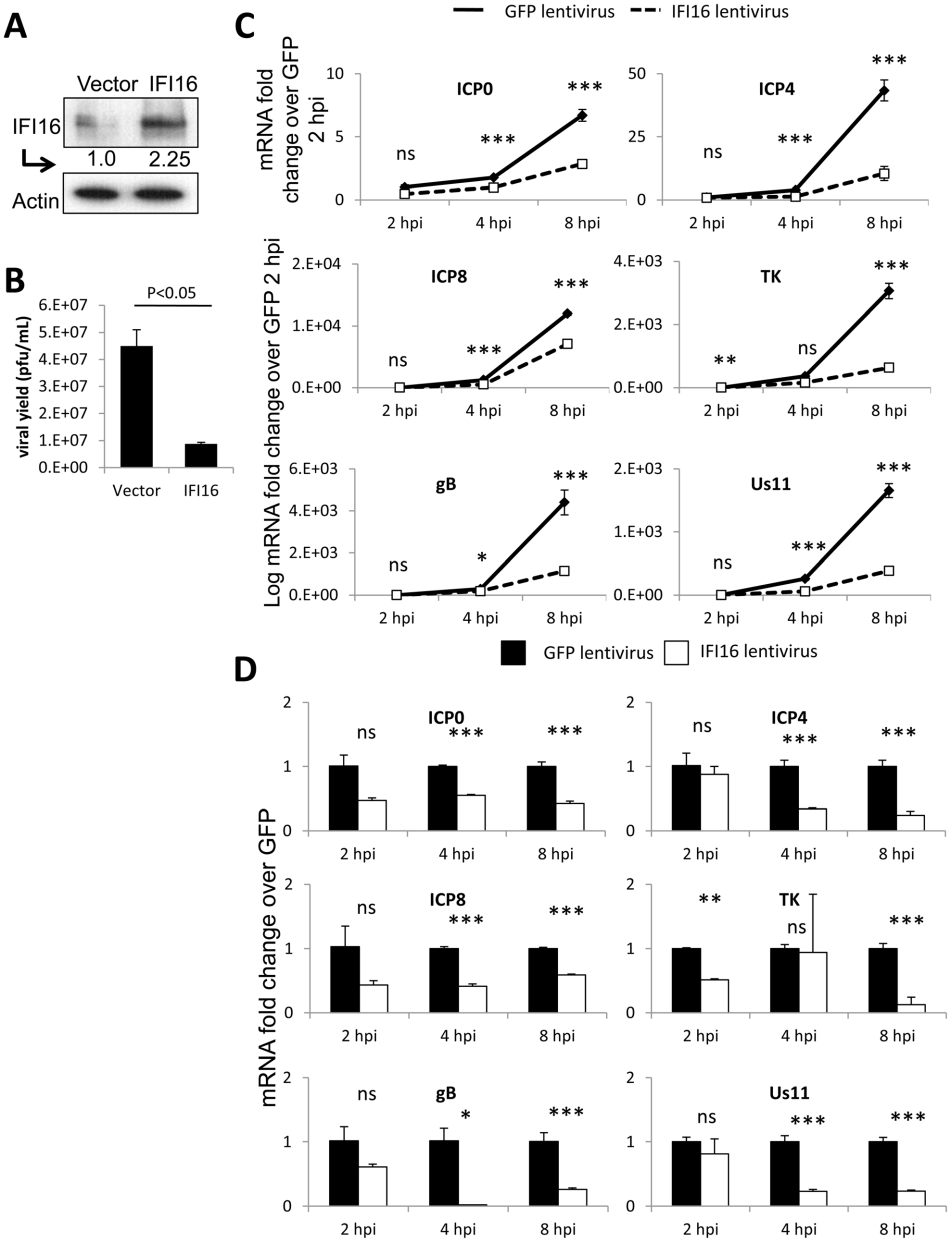
Effect of IFI16 overexpression on HSV-1 gene expression, replication and viral yield in U2OS cells. Lentivirus with the coding region of IFI16 or GFP was transduced into U2OS cells. (A) Western blot analysis of IFI16 expression, 48 h post transduction. Actin is a loading control. (B) Viral yield at 24 h p.i. from cells originally infected at an moi of 0.1 pfu/cell. (C) Representative HSV-1 gene expression from cells infected at an moi of 1 pfu/cell. Shown are mRNA fold-change over siCtrl at 2 h p.i. (C) or concurrent siCtrl samples (D) normalized to GAPDH, ± standard deviation. Statistics were done using Student's T test (ns: not significant, *p<0.05, **p<0.005, ***p<0.0005).

The role of innate immune proteins can be cell-type specific [47]. To determine if our observed effects of IFI16 on HSV-1 replication and gene expression, shown in figure 1 for HFF cells, are also cell-type specific and if over-expression of IFI16 restricts HSV-1 replication and gene expression, we transduced human osteosarcoma U2OS cells with IFI16, which was expressed 2.25-fold over GFP-transduced cells (Figure 2A). HSV-1 yield, measured as in figure 1, was significantly inhibited by >5-fold in U2OS cells overexpressing IFI16 (Figure 2B). HSV-1 gene expression was determined and normalized as above. Consistent with the IFI16 knockdown experiments (Figure 1), though expression of each gene increased over time regardless of the presence of overexpressed IFI16 (Figure 2C), IFI16 overexpression significantly inhibited expression of HSV-1 genes from all gene classes over the course of 8 h of infection: compared to GFP-transduced cells, in IFI16-transduced cells at 8 h p.i., ICP0 was inhibited by >50%, ICP4 was inhibited by 76%, ICP8 was inhibited by 50%, TK was inhibited by 87%, gB was reduced by 74%, and Us11 was inhibited by 77% (Figure 2D).

**Figure 3:**
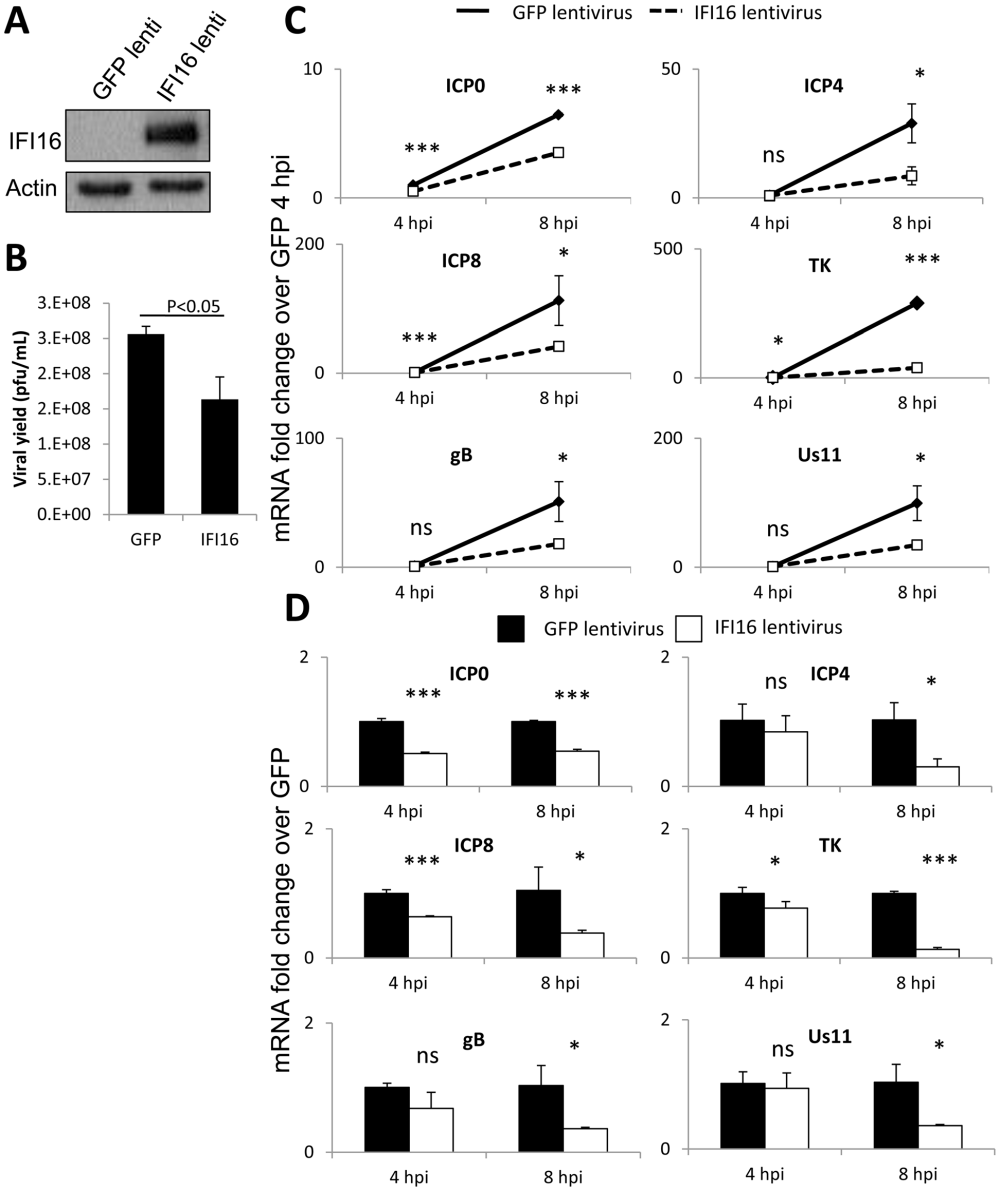
Effect of IFI16 overexpression on HSV-1 gene expression, replication and viral yield in MCF7 cells. Lentivirus encoding IFI16 or GFP was transduced into IFI16-negative MCF7 cells. (A) Western blot analysis of IFI16 expression at 48 h post transduction. Actin is a loading control. (B) Viral yield at 24 h p.i. from cells originally infected at an moi of 0.1 pfu/cell. (C) Representative HSV-1 gene expression from cells infected at an moi of 1 pfu/cell. Shown are mRNA fold-change over siCtrl at 2 h p.i. (C) or concurrent siCtrl samples (D), normalized to GAPDH, ± standard deviation. Statistics were done using Student's T test (ns: not significant, *p<0.05, **p<0.005, ***p<0.0005).

Human breast epithelial cancer MCF-7 cells are naturally IFI16-deficient (Figure 3A) [48]. To determine the effect of IFI16 expression on HSV-1 replication and gene expression in these IFI16-negative cells, we transduced MCF-7 cells with lentiviruses expressing IFI16 or GFP (Figure 3A). HSV-1 replication was inhibited ∼1.6-fold by IFI16 overexpression in MCF-7 cells (Figure 3B). Similar to the results observed in U2OS cells, though expression of each HSV-1 gene increased over the course of infection (Figure 3C), viral gene expression was relatively diminished in IFI16-expressing MCF-7 cells over the course of 8 h of infection. In comparison to expression in GFP-transduced, IFI16-negative MCF-7 cells, in IFI16-transduced cells at 8 h p.i., ICP0, ICP4, ICP8, TK, gB, and Us11 were inhibited by 50%, 70%, 62%, 87%, 46%, and 65%, respectively (Figure 3D).

Together, the results shown in figures 1, 2, and 3 confirmed that IFI16 restricts HSV-1 replication, as was first published by Gariano et al. in HFF cells [45], and suggested that the mechanism of restriction is at the level of HSV-1 gene expression and is not cell-type dependent.

### IFI16 restriction of HSV-1 replication is independent of the inflammasome adaptor protein, ASC, and the IFN response

**Figure 4:**
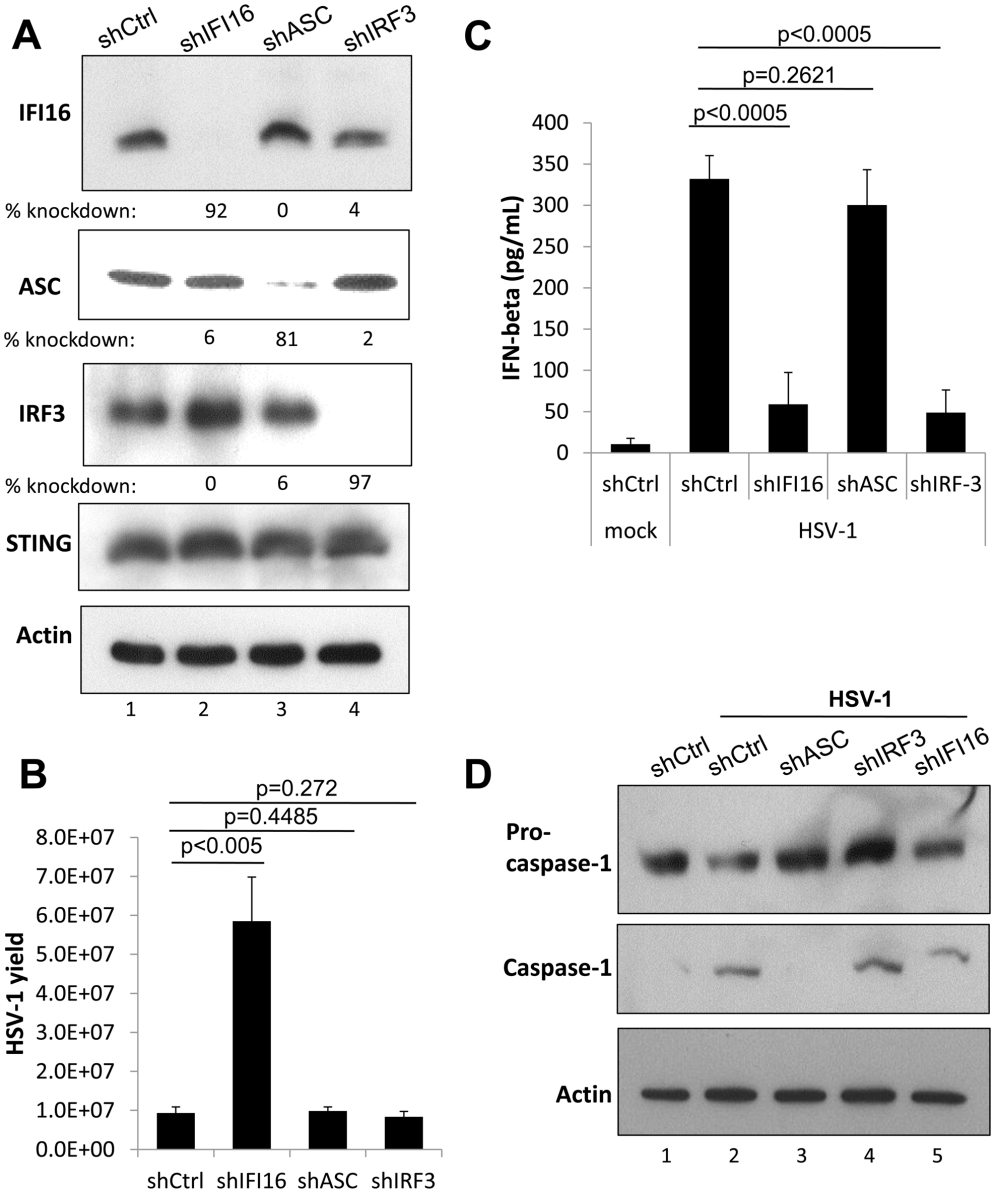
Effect of IFI16, ASC, and IRF3 knockdown on HSV-1 replication. HFF cells were transduced with lentiviruses encoding scrambled shRNA (shCtrl), and shRNA targeting IFI16, ASC, or IRF3. (A) Western blot showing IFI16, ASC, IRF3, and STING, 48 h post transduction, using actin as a loading control. (B) Viral yield at 24 h p.i. from transduced HFF cells originally infected at an moi of 1 pfu/cell, 48 h post transduction. (C) Cell culture supernatant IFNβ levels as determined by ELISA from transduced HFF cells infected at an moi of 1 pfu/cell at 6 h p.i. 48 h post transduction. Statistics were done using Student's T test (ns: not specific). (D) Western blot showing procaspase-1 and caspase 1, from transduced HFF cells infected at an moi of 1 pfu/cell at 6 h p.i., 48 h post transduction, using actin as a loading control.

Recently, we showed that HSV-1 infection induces the IFI16 and NLRP3 inflammasome, causing IFI16 and NLRP3 to associate with the inflammasome adaptor protein, ASC, and resulting in the maturation of caspase-1 and IL-1β [19]. IFI16 also has a role in the expression of HSV-1-induced type I IFN [20]. To determine if the inflammasome or IFN response play roles in the innate inhibition of HSV-1 replication and gene expression, we transduced HFF cells with shRNA-expressing lentiviruses targeting IFI16, ASC, or IRF3 resulting in 92%, 81%, and 97% knockdown of IFI16, ASC, and IRF3, respectively (Figure 4A, lanes 2, 3, and 4, respectively, compared with lane 1). None of the knockdown conditions tested affected STING levels (Figure 4A), showing knockdown specificity. Knockdown of ASC did not affect HSV-1 viral yield, as measured by plaque assay (Figure 4B). Consistent with previous reports showing no effect on wt- or ICP0-null HSV-1 replication in the absence of IRF3 [49], knockdown of IRF3 also did not affect HSV-1 yield (Figure 4B). In contrast, consistent with our previous results, knockdown of IFI16 resulted in a significant >5-fold increase of HSV-1 yield (Figure 4B). To further confirm functional knockout of IFI16 and IRF3, we assayed HSV-1-infected HFF cell culture supernatant IFNβ levels at 6 h p.i., infected 48 h post-transduction at an moi of 1 pfu/cell. A robust IFNβ response was detected from HSV-1-infected cells that had been transduced with shCtrl or shASC but not from cells transduced with shIFI16 or shIRF3 (Figure 4C). To confirm functional knockout of ASC, we determined procaspase-1 cleavage in shRNA-transduced HFF cells infected with HSV-1 at an moi of 1 pfu/cell at 6 h p.i., 48 h post transduction. Procaspase-1 was cleaved to active caspase-1 in HSV-1-infected cells transduced with shCtrl, shIRF3, and, to a lesser extent, with shIFI16, likely due to the HSV-1-induced activation of the nucleotide binding and oligomerization (NOD)-like receptor family pyrin domain-containing 3 (NLRP3) inflammasome [19], but not in mock infected cells or in HSV-1-infected cells transduced with shASC (Figure 4D, compare lanes 1 and 3 with lanes 2, 4, and 5). These studies suggested that the role of IFI16 in the inhibition of HSV-1 viral replication is independent of its role in HSV-1-induced inflammasome activation and interferon induction.

### Cas9-mediated IFI16 gene editing in U2OS cells results in permanent IFI16 protein depletion

**Figure 5:**
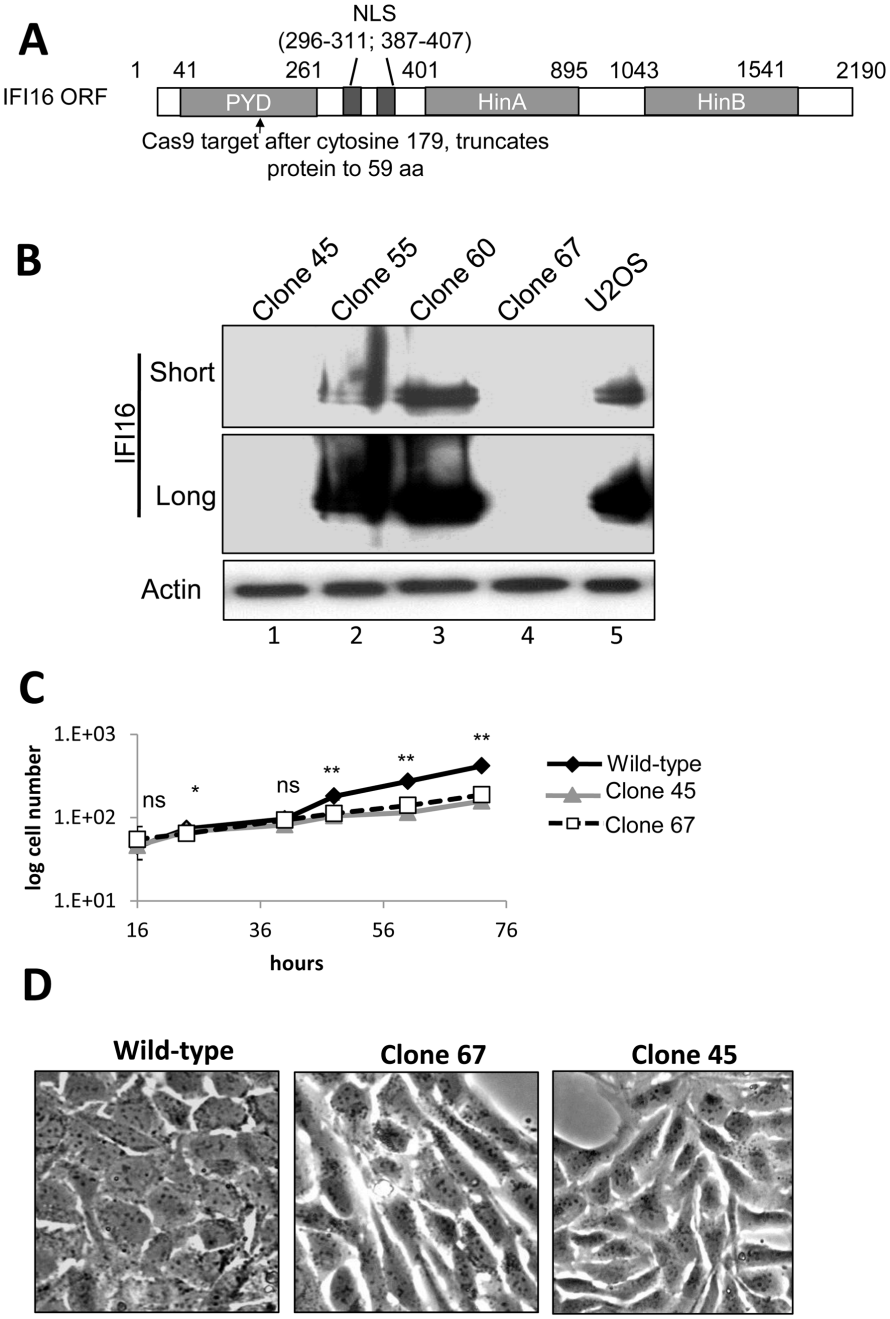
Cas9-mediated IFI16 gene editing and permanent depletion from U2OS cells. IFI16 was depleted from U2OS cells using Cas9 and guided RNA targeting the PYD of IFI16. (A) Schematic representation of Cas9 target site within the 2190 bp IFI16 ORF. NLS: Nuclear localization signal; PYD: Pyrin domain; HinA and HinB: DNA binding domains. (B) Western blot of IFI16 in wt U2OS and a selection of clonal U2OS cells. Short and long exposure of the same IFI16 blot are shown. Actin was used as a loading control. (C) wt-U2OS, clone 45, and clone 67 cell growth. (D) Phase micrograph of wt-U2OS, clone 67, and clone 45 cells at 40×. Statistics were done using Student's T test (ns: not significant, *p<0.05, **p<0.005).

Transfection and transduction of cells leads to activation of innate immune responses and adversely effects HSV-1 replication [50]–[52]. In addition, exogenous DNA introduced into cells activates the absent in melanoma 2 (AIM2) and/or NLRP inflammasome responses [19], [39], [40], [42], [53]. To eliminate potential artifacts from these effects and to more thoroughly investigate the effects of IFI16 on HSV-1 replication and gene expression, we used Clustered Regularly Interspaced Short Palindromic Repeat (CRISPR) Cas9-mediated genome editing [54], a highly specific method for targeted eukaryotic genome editing [55]–[57], to create a permanent IFI16-negative cell line. We designed guided RNA to target the Cas9 endonuclease to a region within the coding sequence for the IFI16 Pyrin domain (PYD), the first functional domain of IFI16 (Figure 5A). Wt U2OS cells were cotransfected with 3 plasmids encoding the guided RNA, Cas9, and GFP (a marker for transfection) at a ratio of 4∶1∶1, respectively. After 48 h, cells were sorted for GFP expression and grown clonally before screening for IFI16 expression by dot blot and western blot (Figure 5B). The IFI16-negative U2OS clones 45 and 67 (Figure 5B, lanes 1 and 4, compared with lane 5) were further characterized.

IFI16-negative U2OS cell growth was moderately slower than that of the wt U2OS parental cells; doubling time for wt cells was approximately 31.8 h and those of clones 45 and 67 were approximately 40 h (Figure 5C). In addition, the deletion of IFI16 in U2OS cells caused a significant change in cellular morphology, leading to rounded, elongated cells when compared with the parental wt cells (Figure 5D). We selected clone 67-U2OS for further experiments.

### HSV-1 replication and gene expression is increased in Cas9-mediated IFI16-negative U2OS cell line

**Figure 6:**
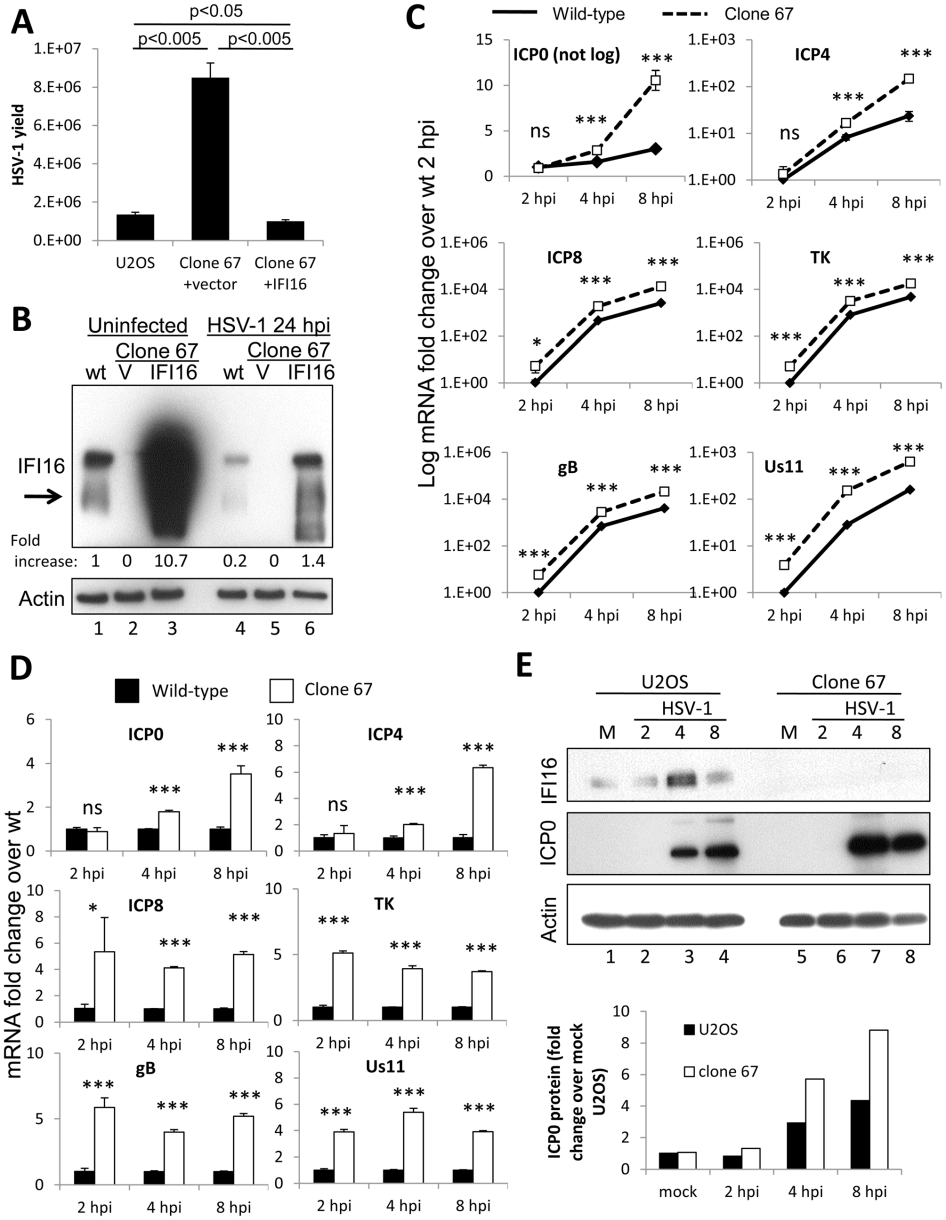
Effect of Cas9-mediated U2OS cell IFI16 gene edition on HSV-1 gene expression and replication, and comparison with wt U2OS cells. (A) HSV-1 yield at 24 h p.i. from wt U2OS cells and clone 67 U2OS cells transduced with empty vector or IFI16 expression vector originally infected at an moi of 0.1 pfu/cell. (B) Western blot of IFI16 and actin in uninfected wt U2OS cells and clone 67 U2OS cells transduced with empty vector (V) or IFI16 expression vector (IFI16), or cells infected with HSV-1 for 24 h at an moi of 1 pfu/cell. (C–D) Representative HSV-1 gene expression from cells infected at an moi of 1 pfu/cell. Shown are mRNA fold-change over GFP at 2 h p.i. (C) or concurrent GFP samples (D), ± standard deviation. Statistics were done using Student's T test (ns: not significant, *p<0.05, **p<0.005, ***p<0.0005). (E) Western blot of IFI16 and ICP0 in U2OS and clone 67 U2OS cells infected with HSV-1 at an moi of 1 pfu/cell for the times indicated (upper panel). Actin is used as a loading control. Densitometric analysis of ICP0 band intensities relative to actin from western blots (lower panel).

To ensure that the stimulatory effects of IFI16 depletion on HSV-1 replication occurred in these newly generated IFI16-negative U2OS cells, we assayed viral yield at 24 h p.i. from wt U2OS, IFI16-negative clone 67, and clone 67 cells transduced with IFI16 to rescue the effects of IFI16 in IFI16-negative cells. When compared with that in parental wt U2OS cells, HSV-1 yield from clone 67 cells was increased over 6-fold in IFI16-negative clone 67 U2OS cells (Figure 6A). Transduction of IFI16 into clone 67 cells resulted in HSV-1 yield 30% lower than that from wt U2OS cells (Figure 6A), consistent with higher levels of IFI16 expression in these cells (Figure 6B), confirming that IFI16 is the factor responsible for HSV-1 restriction and further suggesting specificity of the Cas9-mediated IFI16 deletion in clone 67 cells.

HSV-1 gene expression at 2, 4, and 8 h p.i. was also determined in these cells. Consistent with our experiments with transient knockdown of IFI16, permanent IFI16 depletion led to increased HSV-1 gene expression from all temporal classes of genes over the course of 8 h of infection, though expression of all the genes increased over the course of infection in both conditions (Figure 6C). Compared with that in IFI16-positive parental U2OS cells, in clone 67 cells at 8 h p.i., ICP0 was increased 3-fold, ICP4 was increased 7-fold, ICP8 was increased 5-fold, TK was increased 3-fold, gB was increased 5-fold, and Us11 was increased 4-fold (Figure 6D).

In addition, we examined IFI16 protein levels in wt U2OS cells and clone 67-U20S cells transduced with empty vector or with IFI16 expression vector in the absence of HSV-1 infection or at 24 h p.i. (Figure 6B). Though previous results showed stability of IFI16 in HSV-1-infected U2OS cells up to 12 h [44], IFI16 protein was decreased 80% in wt-U2OS cells (Figure 6B, lane 4 compared with lane 1) and 87% in IFI16-transduced clone 67 cells (Figure 6B, lane 6 compared with lane 3) after a 24 h infection with HSV-1 at an moi of 1 pfu/cell. To ensure IFI16 stability in HSV-1-infected U2OS cells over the course of our gene expression analysis, we performed western blot analysis of HSV-1-infected U2OS and clone 67 cells. IFI16 was stable in U2OS cells up to 8 h p.i. and was, in fact, induced between 2–4 hours (Figure 6E, lane 3 compared with lanes 1–3), consistent with the induction seen in HSV-1-infected HFF cells [19]. ICP0 protein expression, as measured by western blot (Figure 6E, top) and quantified by densitometry (Figure 6E, bottom), was increased in clone 67 cells when compared with that in wt U2OS cells (Figure 6E, compare lanes 7 and 8 with lanes 3 and 4), consistent with our mRNA results (Figure 6C).

Together, these results demonstrated that our permanent IFI16-negative cell line is an appropriate tool to further study the inhibition of HSV-1 replication and gene expression by IFI16.

### IFI16 colocalizes with the HSV-1 genome by 30 min post infection

**Figure 7:**
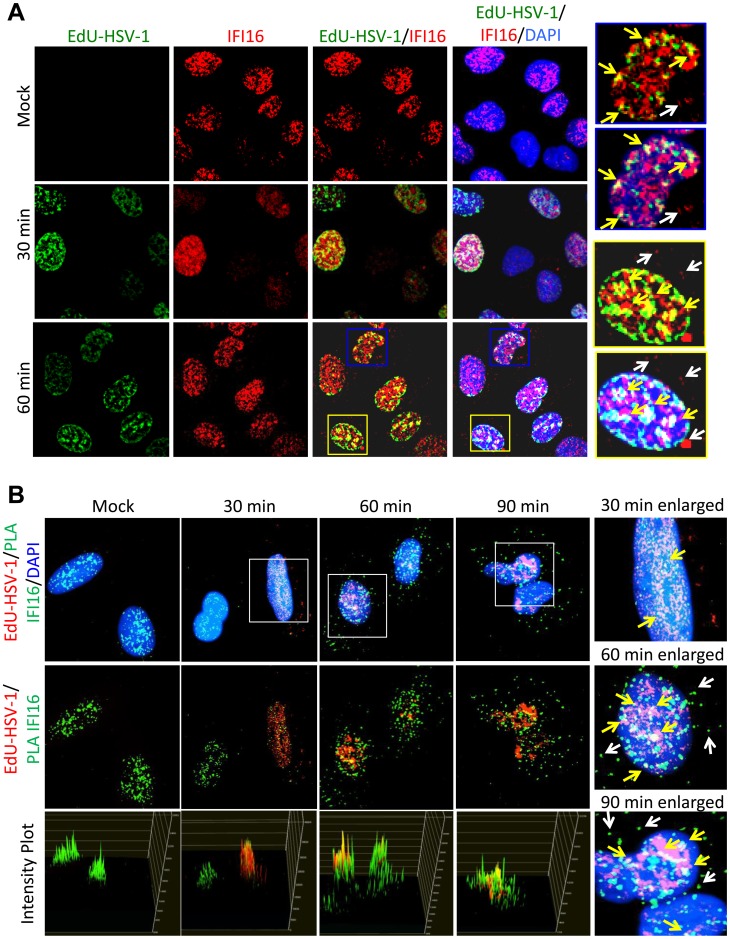
Analysis of HSV-1 genome entry into the nucleus and association with IFI16. U2OS cells were mock infected or infected with EdU-labeled HSV-1 at an moi of 1 pfu/cell for 30 or 60 min. (A) Immunofluorescence analysis of IFI16 (red) and EdU detection of HSV-1 DNA (green). Boxed areas are enlarged on the right. Yellow arrows indicate colocalized IFI16 and HSV-1 DNA. White arrows indicate cytoplasmic IFI16. (B) EdU detection of HSV-1 DNA (red) and PLA for IFI16/IFI16 (green), including intensity plots for red and green channels (lower panels). Boxed areas are enlarged on the right. Yellow arrows indicate colocalized IFI16 and HSV-1 DNA. White arrows indicate cytoplasmic IFI16.

Previously, we showed by FISH analysis and co-immunofluorescence that IFI16 colocalized with the HSV-1 genome at 1 h p.i. in HFF cells [19]. To determine HSV-1 genome recognition by IFI16 and colocalization in U2OS cells, we infected U2OS cells with EdU-labeled HSV-1 for 30 or 60 min, performed immunofluorescence for IFI16, and costained for EdU-labeled HSV-1 genome (Figure 7A). We also performed proximity ligation assays (PLA), a fluorescence-based assay that uses DNA-oligonucleotide-linked secondary antibodies to detect closely associated proteins. If two protein epitopes are within 40 nm of each other, the antibody-linked oligonucleotides can ligate with adaptor oligonucleotides to form complete circles that are replicated via rolling-circle DNA replication and detected with fluorescent sequence-specific probes. PLA provides a method for the detection of weak or transient interactions [58]. It can also provide amplified, very distinct localization of a single protein. Here, we did PLA with two antibodies to IFI16, and costained for EdU-labeled HSV-1 (Figure 7B). In uninfected cells, IFI16 was exclusively nuclear. By 30 min p.i., EdU-HSV-1 was detected in approximately 30% of cell nuclei and colocalized with IFI16 in small nuclear puncta (Figure 7A and B, enlarged images of B, yellow arrows). By 60 min, consistent with previous reports from our laboratory and others [19], [37], some IFI16 was detected in the cytoplasm (Figure 7A and B, white arrows), and nuclear IFI16 largely colocalized with the HSV-1 genome (Figure 7A and B, yellow arrows). Pixel intensity plots for the red and green channels were generated for each of the PLA figures (Figure 7B, bottom panels). These show quite clearly the yellow signals that indicate colocalization between IFI16 (green) and HSV-1 genomic DNA (red). These data are the first to distinctly show IFI16 colocalization with the HSV-1 genome at such early times post-infection in U2OS cells and suggest that the association of IFI16 with HSV-1 occurs very shortly after viral DNA enters the nucleus.

### IFI16 binds HSV-1 genomic DNA close to the transcriptional start sites (TSS) of all gene classes

**Figure 8:**
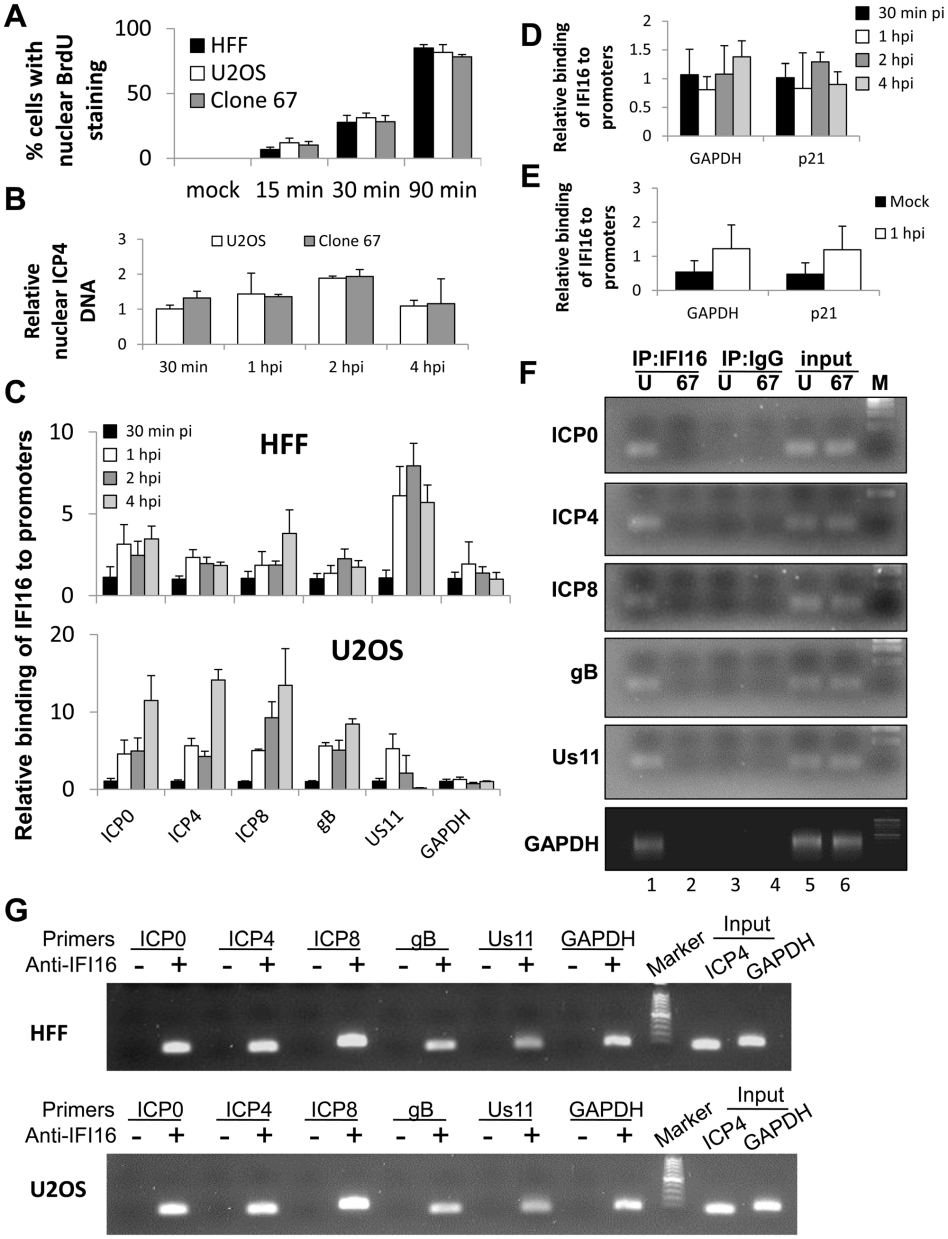
ChIP analysis of IFI16 binding to the HSV-1 genome at the transcriptional start site of viral genes. (A) HFF, U2OS, and clone 67 cells were infected with BrdU-labeled HSV-1 at an moi of 1 pfu/cell for 15, 30, or 90 min before fixation. Cells were immunostained for BrdU. The percent of cells with nuclear BrdU was quantified by scoring BrdU localization in 4 fields of cells per sample (total 150–200 cells/sample). (B–G) U2OS and clone 67 cells were mock infected or infected with wt HSV-1 at an moi of 1 pfu/cell. (B) The relative quantity of nuclear HSV-1 DNA at various times post infection was quantified by qPCR of HSV-1-infected nuclear extracts from U2OS and clone 67 U2OS cells using primers for the ICP4 start site. (C) ChIP analysis of IFI16 in HFF and U2OS cells. IFI16 was immunoprecipitated from cells infected with HSV-1 (1 pfu/cell) for 30 min, 1, 2, or 4 hours. Bound DNA was analyzed by real-time PCR with primers to regions flanking the transcriptional start sites of the genes indicated. Values were normalized to IFI16-bound GAPDH and input ICP4. (D) ChIP analysis of IFI16 binding the cellular promoters for GAPDH and p21 in U2OS cells, performed as above. (E) ChIP analysis of IFI16 binding cellular promoters in uninfected U2OS cells or cells infected with HSV-1 at an moi of 1 pfu/cell for 1 hour, performed as above. (F) Agarose gels showing HSV-1 and cellular promoter DNA precipitated with antibody to IFI16 or control IgG in U2OS (U) or clone 67 (67) cells at 1 h p.i., and an moi of 1 pfu/cell. (G) Agarose gels showing the amplification of the indicated HSV-1 and cellular promoter regions after IFI16 ChIP at 4 h p.i.

HSV-1 gene expression occurs in the nuclei of host cells [1]. To determine if the different morphologies of HFF, U2OS, and clone 67 U2OS cells affected the time post-infection that HSV-1 genomic DNA enters host cell nuclei, we mock infected or infected HFF (Figure S1A), U2OS (Figure S1B), or clone 67-U2OS (Figure S1C) cells with HSV-1 genome-labeled with 5-bromo-2-deoxyuridine (BrdU) at an moi of 1 pfu/cell for 15, 30, or 90 min and immunostained for BrdU. Cytoplasmic BrdU staining showed punctate spots, likely representing nucleocapsid-bound HSV-1 genomes [1]. BrdU-HSV-1 genomes first appeared in the nuclei of approximately 5% of all three cell types at 15 min p.i. (Figure 8A). Once in the nucleus, BrdU staining was still somewhat punctate in many cells but was, overall, much more diffuse than cytoplasmic BrdU staining, likely reflecting the relative expansion of nuclear HSV-1 DNA compared with that condensed in nucleocapsids, consistent with previous studies [59], [60]. The percent of cells with nuclear BrdU increased to approximately 25% at 30 min and to 80% at 90 min p.i. These results demonstrated that the kinetics by which the HSV-1 genome enters host cell nuclei is consistent between HFF, U2OS, and clone 67-U2OS cells in that the genome begins to enter host cell nuclei within 15 min of exposure to the virus, and nuclear HSV-1 DNA increased steadily in a consistently measurable proportion of cells at 30 min p.i. These data suggest that the kinetics of HSV-1 genome entry into cell nuclei is not affected by the morphology of these cell types.

Because the number of nuclei with HSV-1 changed over time, we more quantitatively determined the relative nuclear HSV-1 levels by qPCR of the ICP4 promoter region in nuclear extracts of HSV-1-infected U2OS and clone 67 cells from 30 min to 4 h p.i., and normalized to GAPDH and to DNA levels in U2OS cells at 30 min p.i. using the ddCt method (Figure 8B). We observed that there was variation in both U2OS and clone 67-U2OS cells over time, consistent with previous studies [13], but no difference between cell types at any time (Figure 8B). Based on these results, we used 30 min p.i. as the earliest time point in all our subsequent HSV-1 gene expression studies.

IFI16 binds the sugar-phosphate backbone of DNA in a purportedly sequence-independent manner *in vitro* [61] and has been shown to interact with oligonucleotides derived from HSV-1 [28], [37] and colocalize with HSV-1 in infected cell nuclei (Figure 7) [19]. There is some preference for cruciform or super helical DNA *in vitro* [29], but otherwise, little is known about the location of IFI16 binding DNA sites on viral or cellular genomes and no studies have shown, by chromatin immunoprecipitation (ChIP), association of endogenous IFI16 with HSV-1 genomic DNA during infection. Our results in Figures 1, 2, 3, 4, and 6 showed that IFI16 reduced HSV-1 gene expression. We therefore chose to examine its presence at the transcriptional start sites (TSS) of HSV-1 genes.

To determine whether IFI16 binds HSV-1 gene TSS, we performed ChIP analysis of HSV-1 infected cells. IFI16 was immunoprecipitated from the nuclei of HFF and wt-U2OS cells infected with HSV-1 (1 pfu/cell) for 30 min, 1, 2, or 4 h. DNA associated with IFI16 was analyzed by qPCR with primers corresponding to regions flanking the TSS of HSV-1 ICP0, ICP4, ICP8, gB, and Us11 genes, as well as the cellular GAPDH and p21 genes. Because levels of nuclear HSV-1 DNA change up to 2-fold over the course of infection, we normalized our ChIP data concerning HSV-1 DNA to input HSV-1 (ICP4 promoter region) to avoid artifacts from the different sample inputs. Cellular gene DNA was normalized to input GAPDH. For each promoter tested data were further normalized to the 30 min time point using the ddCt method [46].

IFI16 antibodies coprecipitated each of the viral TSS tested in both HFF and wt-U2OS cells (Figure 8C). The association of IFI16 with these regions increased over the course of the infection for nearly all of the genes in both cell types, most dramatically between 30 min and 1 h p.i. Interestingly, in HFF cells, IFI16 accumulated most heavily onto the Us11 promoter whereas it accumulated least on the Us11 promoter in U2OS cells (Figure 8C).

In an earlier study, IFI16 was not found to be bound to cellular promoters in HCMV infected HFF cells [43]. However, we found IFI16 bound to the GAPDH and p21 promoters in HFF and U2OS cells that were mock infected or infected with HSV-1 (Figure 8D and E). This association did not significantly change between mock infected or HSV-1 infected cells or over the course of the 4-hour HSV-1 infection (Figure 8D and E).

To ensure specificity of our ChIP assays, we performed additional ChIP analysis of HSV-1-infected U2OS (U) or clone 67 U2OS (67) cells with IFI16 antibody or control IgG. Promoter sequences were amplified and analyzed by agarose gel electrophoresis. IFI16 antibodies, but not control IgG, precipitated viral and cellular promoters from wt U2OS cells but not IFI16-negative clone 67 U2OS cells (Figure 8F, compare lane 1 with lanes 2–4). Importantly, there was no amplification of DNA from these regions in no-antibody controls at 4 h p.i. (Figure 8G).

These data are the first to show endogenous IFI16 binding to HSV-1 DNA during infection and suggested that IFI16 targets all temporal classes of HSV-1 genes and does not specifically affect the immediate early and early regulatory genes.

### IFI16 inhibits association of RNA polymerase II with HSV-1 transcriptional start sites

**Figure 9:**
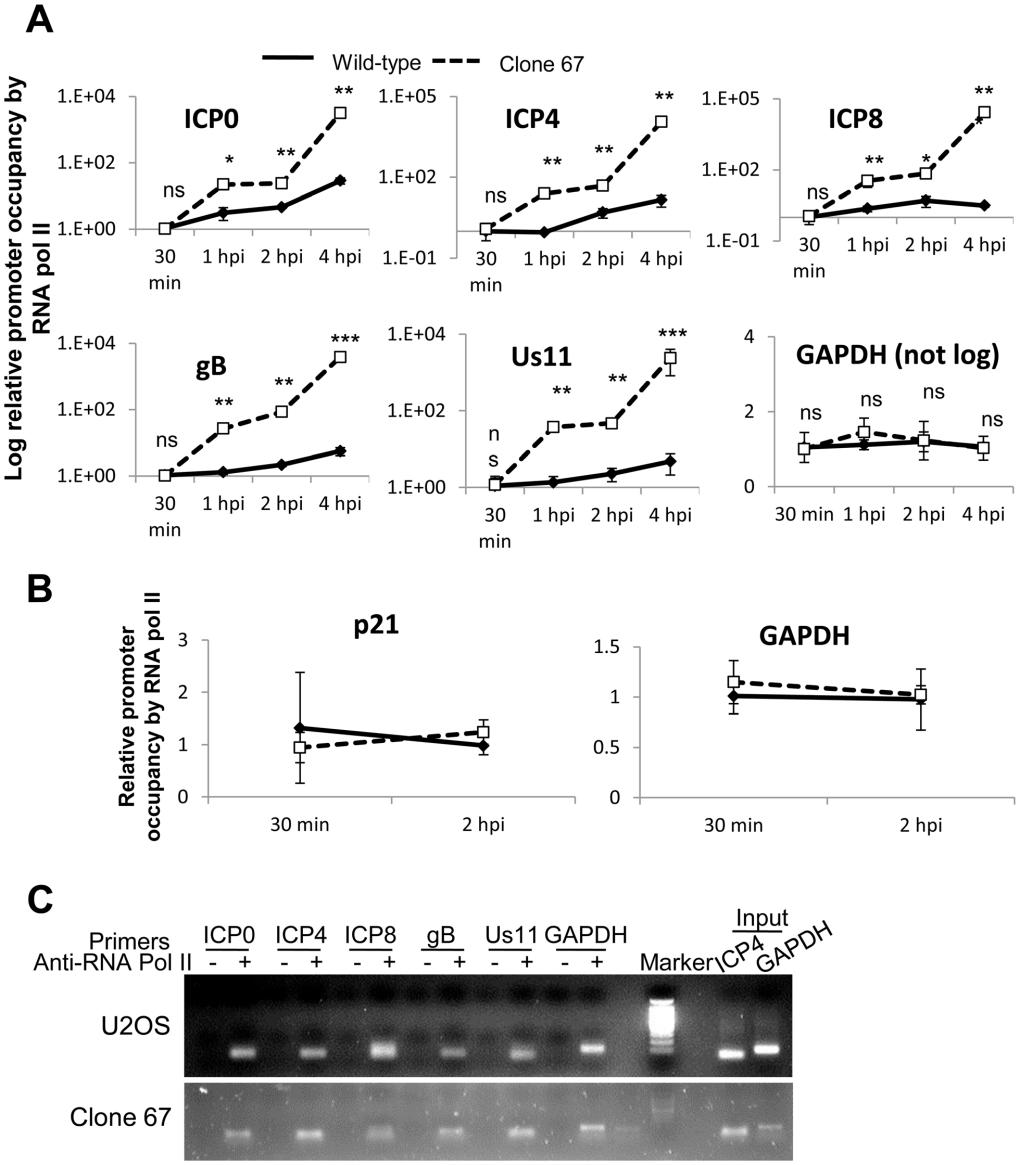
IFI16 prevents RNA Pol II accumulation at HSV-1 promoters. U2OS and clone 67 cells were infected with HSV-1 at an moi of 1 pfu/cell for 30 min, 1, 2, or 4 h. ChIP analysis was done with RNA pol II antibody and primers to the indicated HSV-1 genes, normalized to input ICP4, and cellular promoters normalized to GAPDH and are shown as relative to the association of RNA pol II and the indicated promoter at 30 min p.i. (A and B). Statistics were done using Student's T test (ns: not significant, *p<0.05, **p<0.005, ***p<0.0005). (C) Representative amplification products of the indicated promoters precipitated with beads alone or antibody to RNA polymerase II at 4 h p.i.

HSV-1 genes are transcribed by cellular RNA pol II [1]. Our data show that IFI16 binds HSV-1 DNA (Figure 8). To determine whether the presence of IFI16 affects the accumulation of RNA pol II on the various HSV-1 TSS, we infected wt and clone 67 U2OS cells with HSV-1 at an moi of 1 pfu/cell for 30 min, 1, 2, or 4 h and performed ChIP analysis with an antibody to RNA pol II and primers to HSV-1 TSS, as described above, normalizing once again to input ICP4 levels because of the differences observed in nuclear HSV-1 DNA levels (Figure 8B). RNA pol II accumulation at HSV-1 TSS was increased significantly in IFI16-negative cells for all viral genes tested compared with that in parental U2OS cells (Figure 9A). Interestingly, RNA pol II accumulation at the cellular GAPDH gene promoter was not affected by the presence or absence of IFI16 (Figure 9A). To confirm that RNA pol II association was unchanged on cellular promoters in the presence or absence of IFI16, we repeated the RNA pol II ChIP, in U2OS and clone 67 cells that were infected with HSV-1 for 30 min or 2 h, with primers for the GAPDH and p21 promoter regions. RNA pol II association with each of these regions was the same, regardless of infection time or the presence of IFI16 (Figure 9B). HSV-1 and GAPDH amplicons were detected in samples from U2OS and clone 67 cells immunoprecipitated with RNA pol II but not with beads alone at 4 h p.i. (Figure 9C).

These results suggested that the presence of IFI16 restricts the association of RNA pol II with HSV-1 TSS of all temporal classes, consistent with IFI16-mediated HSV-1 gene repression (Figures 1–3).

### IFI16 inhibits association of transcription factors, TATA binding protein (TBP) and Oct 1, with HSV-1 transcriptional start sites

HSV-1 gene expression is dependent on many viral and cellular transcription factors [1], [59], [62]–[64]. Some of these factors, including the TATA binding protein (TBP) and Oct1 are necessary to recruit RNA pol II to all HSV-1 promoters, in the case of TBP, and only to immediate early promoters, in the case of Oct1 [65], [66]. Because IFI16 has been shown to prevent the association of some transcription factors with the HCMV promoter [45], we hypothesized that IFI16 prevents association of TBP and Oct1 with HSV-1 promoters, thereby inhibiting the recruitment of RNA pol II for gene expression that we observed (Figure 9).

**Figure 10:**
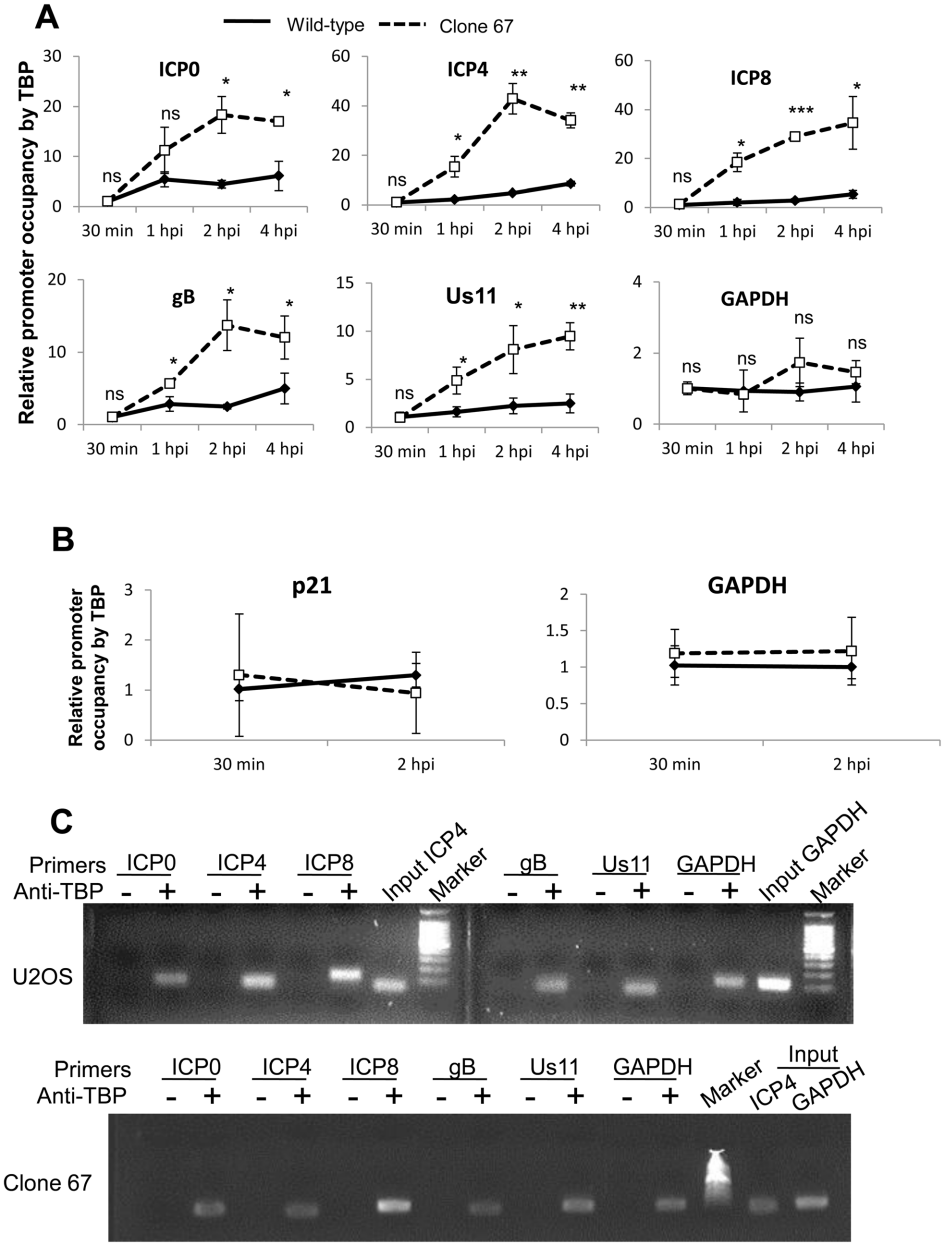
IFI16 prevents TBP accumulation at HSV-1 promoters. Wt U2OS and clone 67 U2OS cells were infected with HSV-1 at an moi of 1 pfu/cell for 30 min, 1, 2, or 4 h. ChIP analysis was done with TBP antibody and real-time PCR primers to the indicated HSV-1 genes, normalized to input ICP4 for viral promoters and to GAPDH for cellular promoters, and are shown as relative to the association of TBP and the indicated promoter at 30 min p.i. (A and B). Statistics were done using Student's T test (ns: not significant, *p<0.05, **p<0.005, ***p<0.0005). (C) Representative amplification products of the indicated promoters precipitated with beads alone or antibody to TBP at 4 h p.i.

To determine whether IFI16 reduces the association of TBP with HSV-1 promoters, we infected wt and clone 67 U2OS cells with HSV-1 at an moi of 1 pfu/cell for 30 min, 1, 2, or 4 h, performed ChIP analysis with antibodies to TBP and primers to HSV-1 TSS (Figure 10), and normalized as described above. The absence of IFI16 expression led to significantly increased (3- to 10-fold) association of TBP with HSV-1 promoters but no change in association of TBP with the GAPDH promoter (Figure 10A). To confirm that TBP association with cellular promoters was unchanged by the presence of IFI16, we repeated the TBP ChIP in both cell types infected with HSV-1 for 30 min or 2 h, and amplified the GAPDH and p21 TSS. TBP association with each of these regions was unchanged, regardless of infection time or the presence of IFI16 (Figure 10B). DNA was amplified only from samples from both cell types immunoprecipitated with antibodies to TBP but not from beads alone controls (Figure 10C).

**Figure 11:**
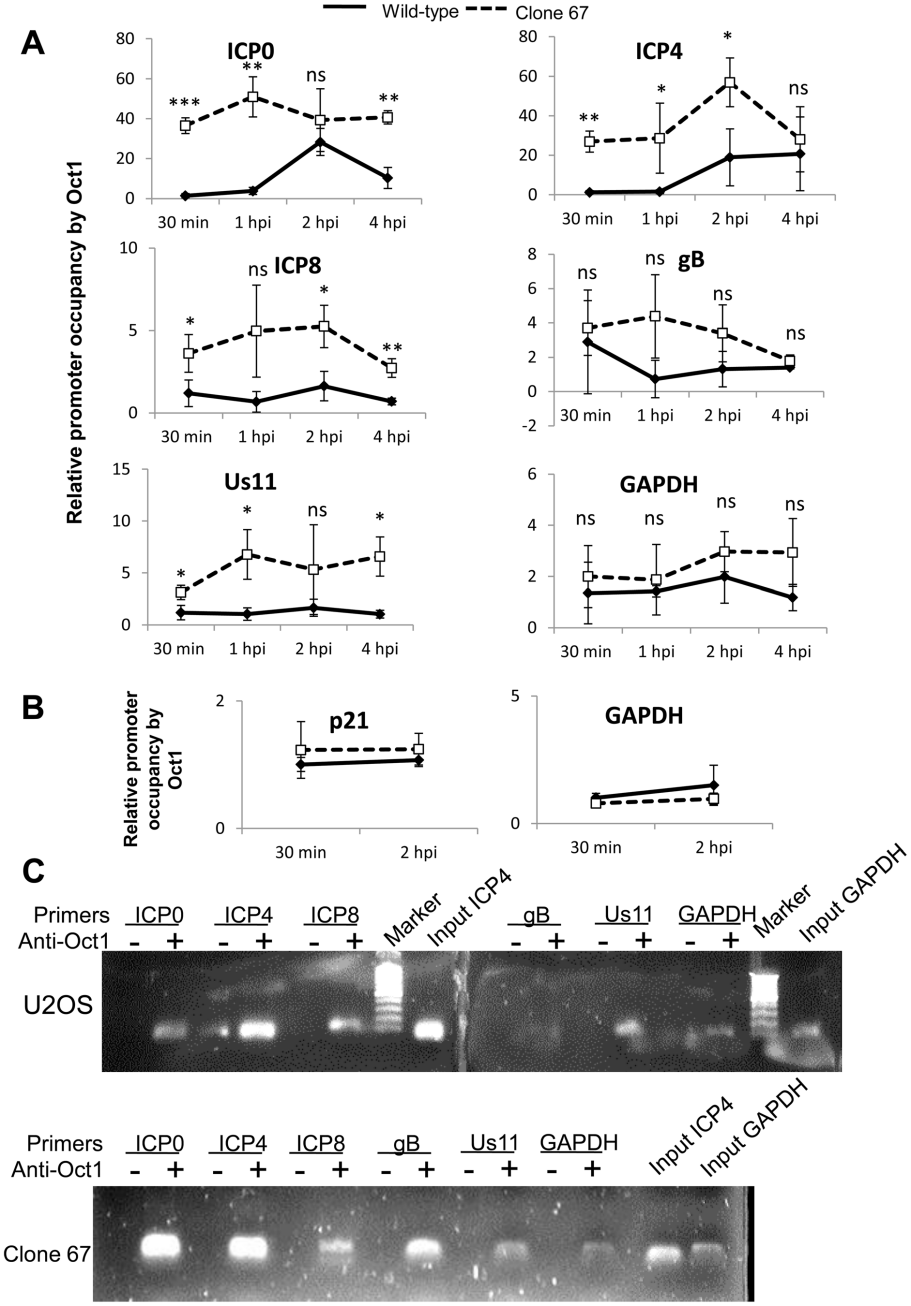
IFI16 prevents Oct1 accumulation at HSV-1 promoters. U2OS and clone 67 cells were infected with HSV-1 at an moi of 1 pfu/cell. ChIP analysis was done with Oct1 antibody and real-time PCR with primers to the indicated HSV-1 genes, normalized to input ICP4, and cellular promoters normalized to GAPDH and are shown relative to the association of Oct1 and the indicated promoter in U2OS cells. Statistics were done using Student's T test. (ns: not significant, *p<0.05, **p<0.005, ***p<0.0005) (A and B). (C) Representative amplification products of the indicated promoters precipitated with beads alone or antibody to Oct1 at 4 h p.i.

To determine if IFI16 reduces association of Oct1 with HSV-1 promoters, we infected wt and clone 67 U2OS cells with HSV-1 at an moi of 1 pfu/cell for 30 min, 1, 2, or 4 h and performed ChIP analysis with antibodies to Oct1 and primers to HSV-1 TSS as described above. The absence of IFI16 expression led to significantly increased (3- to 17-fold) association of Oct1 with HSV-1 immediate early ICP0 and ICP4 promoters, a very modest increase in association of Oct1 with ICP8 and Us11 promoters, and no significant change in the association of Oct1 with gB or GAPDH promoters (Figure 11A). To confirm that Oct1 association with cellular promoters was not altered by the presence or absence of IFI16, we repeated the Oct1 ChIP in both cell types infected with HSV-1 for 30 min or 2 h, with primers for GAPDH and p21 promoters. Oct1 association with each of these regions was the same, regardless of infection time or the presence of IFI16 (Figure 11B). Amplified DNA was observed only from samples immunoprecipitated with antibodies to TBP but not in the control beads alone samples at 4 h p.i. (Figure 11C).

Together, these results demonstrated that IFI16 does, indeed, prevent and/or reduce association of transcription factors with HSV-1 promoters but not cellular promoters and provide a potential explanation for the decrease in RNA pol II association with HSV-1 promoters in IFI16 positive cells (Figure 9).

### IFI16 induces changes in markers for heterochromatin and euchromatin on viral and cellular promoters

Orzalli et al. [38] suggested that IFI16 may induce changes in histone modifications associated with HSV-1 promoters in HFF cells at 6 h p.i. during infection with an ICP0-null virus, but saw no effect on histone modification with their rescue virus. Because we observed IFI16-induced differences in HSV-1 gene expression and replication during infection with wt virus, we hypothesized that there were IFI16-induced differences in the histone modifications associated with wt HSV-1 DNA.

To determine if IFI16 affected the histone modifications in chromatin associated with HSV-1 TSS, we infected wt and IFI16-negative clone 67 U2OS cells with HSV-1 at an moi of 1 pfu/cell for 30 min, 1, 2, or 4 h and performed ChIP analysis with antibodies to total histone H3, H3K4me3 (a marker for active or euchromatin), or H3K9me3 (a marker for repressive or heterochromatin), primers for HSV-1 TSS, and normalized to input ICP4 TSS levels (for HSV-1 TSS) or GAPDH (for cellular TSS), as described above.

**Figure 12:**
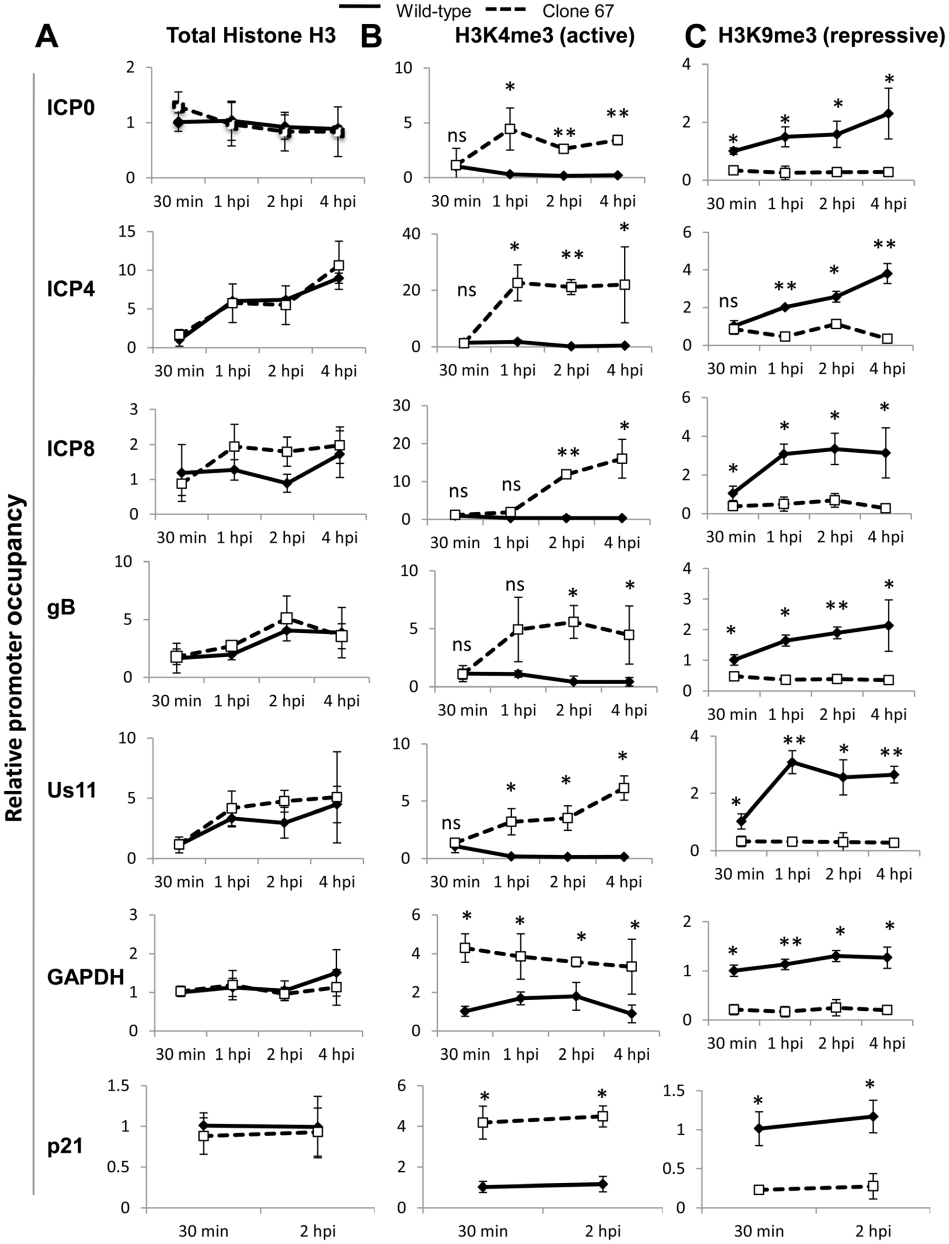
IFI16 induces changes in histone modifications at HSV-1 promoters. Wt U2OS and clone 67 U2OS cells were infected with HSV-1 at an moi of 1 pfu/cell for 30 min, 1, 2, or 4 h. ChIP analysis was done with antibodies to total histone H3 (A), H3K4me3 (B), or H3K9me3 (C) and primers to the indicated genes, normalized to input ICP4 for HSV-1 genes and to input GAPDH for cellular genes and are shown relative to the association of total histone or modified histone at the indicated promoter at 30 min p.i. in each cell type. Statistics were done using Student's T test (ns: not significant, *p<0.05, **p<0.005, ***p<0.0005).

As determined previously [38], the presence of IFI16 had no effect on the levels of total histone H3 associated with HSV-1 promoters or the cellular GAPDH or p21 promoters over the course of infection (Figure 12A).

In the presence of IFI16, H3K4me3 (active chromatin) was detected associated with IE, E, and L HSV-1 promoters at 30 min p.i. This association decreased 3- to 5-fold by 4 h p.i. (Figure 12B). At 30 min p.i., there was no effect of IFI16 depletion on H3K4me3 association with viral promoters (Figure 12B). In contrast, IFI16 depletion led to a marked increase in the association of the active H3K4me3 with HSV-1 promoters at later times p.i. (Figure 12B). Interestingly however, the absence of IFI16 also led to an increase in the levels of active H3K4me3 on the GAPDH and p21 promoters (Figure 12B).

IFI16 also had little to no effect on the association of H3K9me3 (repressive chromatin) with IE, E, and L HSV-1 promoters at the very early times post infection but at later times, the presence of IFI16 led to increased association of the repressive chromatin marker with HSV-1 promoters (Figure 12C). In the absence of IFI16, HSV-1 promoter occupancy by H3K9me9 did not change significantly over the course of the 4 hour infection. Again, interestingly, the association of H3K9Me3 with the cellular GAPDH and p21 promoters was increased in the presence of IFI16 at all times p.i. (Figure 12C).

These data corroborate the previous findings that IFI16 does not affect the association of total histones with HSV-1 DNA [38] but demonstrated that there is a global decrease in repressive heterochromatin markers and an increase in active euchromatin markers associated with wt HSV-1 and cellular promoters in the absence of IFI16, which was not observed in previous studies.

## Discussion

**Figure 13:**
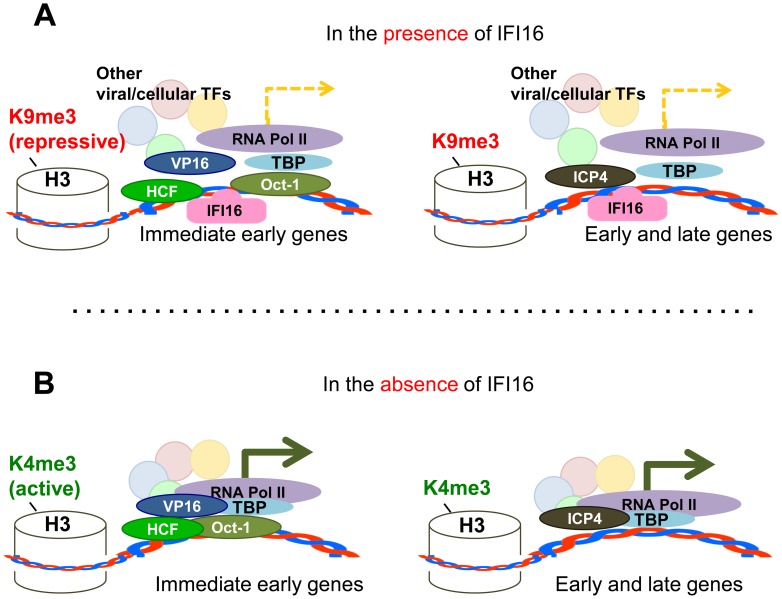
Schematic model: IFI16 inhibits HSV-1 gene expression by modulating histone modifications and binding to HSV-1 promoters, specifically preventing or decreasing the association of transcription factors at viral promoters. In the presence of IFI16 (A) markers for heterochromatin are loaded onto HSV-1 gene transcription start sites and transcription factors including TBP, RNA pol II, and (in the case of IE genes) Oct1 associate with HSV-1 promoters to stimulate gene expression at low levels. In the absence of IFI16 (B), IFI16 and markers for euchromatin are loaded onto HSV-1 gene transcription start sites, and association of transcription factors with viral gene promoters is increased, thereby significantly increasing viral gene expression.

IFI16 has several antiviral properties, including roles in IFN**β** induction [20], [28], [67], activation of the inflammasome response to nuclear viral DNA [19], [39]–[41], [68], [69], and modulation of HCMV replication and gene expression [45], [70]. In this study, we show that HSV-1 replication and gene expression are inhibited by IFI16. Our data lead us to propose a model whereby, in the presence of IFI16, histone H3 associated with viral and cellular genes is modified by repressive trimethylation at lysine 9 and important transcription factors, including TBP and Oct1 (in the case of immediate early genes), bind to HSV-1 transcription start sites, along with IFI16, recruiting RNA pol II to induce transcription at low levels (Figure 13A). However, in the absence of IFI16, there is a global shift from repressive chromatin markers to the active trimethylation at lysine 4 and binding of the transcription factors and, therefore, RNA pol II, to viral promoters is increased (Figure 13B), thereby significantly increasing gene expression.

Interestingly, the repressive effect of IFI16 on wt HSV-1 observed here and previously [45] was not observed for an ICP0 rescue virus [38] or wt HSV-1 strain 17 [44]. To a point, similar methodologies were used for all: plaque assay to determine HSV-1 replication in fibroblasts depleted for IFI16 by sh- or siRNA. Here, we show derepression of HSV-1 replication and gene expression HFF and U2OS cells using siIFI16, shIFI16, and CRISPR technology to delete IFI16. We also show repression of HSV-1 replication and gene expression in U2OS and MCF7 cells after overexpression of IFI16. Currently, it is difficult to resolve these differences. We suspect that nuanced and dynamic interplay between IFI16 and ICP0 is involved in the repression of HSV-1. Further studies are necessary to fully understand these nuances.

### The role of IFI16 in HSV-1 transcription regulation

Though IFI16 is known to be involved in transcriptional regulation [32]–[34], [48], [70]–[76], the mechanisms of this regulation have been largely undetermined. IFI16 may modulate transcription through association with transcription factors and/or blocking their association with promoters [33], [34], [45], [48], [70], [72]–[74] or it may promote the formation of heterochromatin on promoters, resulting in their repression [38], [76].

Here, we show evidence for both effects during HSV-1 infection; IFI16 reduces association of RNA pol II, TBP, and Oct1 specifically with HSV-1 promoters and not with the promoters of the cellular genes, GAPDH and p21 (Figures 9–11). This suggests that IFI16 can specifically prevent association of transcription factors with HSV-1 DNA while allowing them to associate with cellular genomic DNA.

We also show evidence that IFI16 directly or indirectly impacts histone modifications at the TSS of both HSV-1 and cellular genes (Figure 12). Interestingly, there was an increase in total histone association with only the TSS of ICP4 (Figure 12A). This could be due to the necessity for high levels of ICP4 early during infection to stimulate E and L gene expression and the relatively little ICP4 needed at later times. Additionally, at 30 minutes p.i., there was little difference in histone modifications between cell types, but the increase in H3K4me3 and decrease in H3K9me3 at HSV-1 TSS in IFI16-negative cells compared with that in IFI16-positive cells suggests that these modifications are being added to virally-associated histones largely between 30 min and 1 h p.i. rather than histones being modified prior to association with viral DNA. This alteration of the histone modifications at extremely early times post-infection could be due to ICP0, VP16, IFI16, or other viral or cellular factors, or a combination.

Previous studies have suggested that IFI16 promotes the formation of heterochromatin and reduces the formation of euchromatin on the promoters of an HSV-1 mutant genome, which does not express the viral E3 ubiquitin ligase, ICP0 [38]. However, that study did not show a change in chromatin markers associated with their wt-like rescue virus and did not examine the association of these markers with cellular genes [38]. The difference between our findings and the earlier study is likely due to their normalization of data to GAPDH promoters associated with specific chromatin markers and our normalization to input viral genomes. Normalizing our data to co-precipitated GAPDH sequences would also yield results suggesting no difference between chromatin markers on wt HSV-1 genomes because we found that association of these histone modifications with cellular DNA was also altered in the presence of IFI16 (Figure 12). We postulate that IFI16 may have a global effect on the formation of hetero- and euchromatin and not an effect strictly on viral genome-associated chromatin. This is consistent with previous studies linking IFI16 with histone modifications and histone modification machinery [37], [76] and suggests that IFI16 may play a direct or indirect role in modulating the activity of these enzymes or their association with HSV-1 genes. The role of ICP0 in chromatin remodeling [13] and possible nuclear interaction between IFI16 and ICP0 [19] further suggest a role for IFI16 in histone modification. Further studies are required to fully understand the involvement of IFI16 in chromatin remodeling complexes in the basal state or during infection with HSV-1 or other pathogens.

We found IFI16 associated with GAPDH promoters in HFF cells and U2OS cells (Figure 8). However, previously Li et al. showed that, during HCMV infection, IFI16 associated with viral genes but did not associate with cellular genes [43]. This discrepancy could be due to differences in ChIP protocol; we immunoprecipitated IFI16 from cell nuclear extracts, which reduces background. Li et al., immunoprecipitated from total cell lysate [43], which would lack such nuclear enrichment and, because IFI16 is exported from the nuclei to the cytoplasm of herpesvirus-infected cells [19], [37], [39], [40], [42], [77], immunoprecipitating from total cell lysate could capture ligands associated with cytoplasmic IFI16, possibly reducing the threshold of detection for nuclear associations. However, given that Li et al., show exclusively nuclear IFI16 at the time of their IFI16 ChIP experiments, differences in gene detection by ChIP may be based on the proximity of the primer-amplified DNA region with the binding site of IFI16.

It is also remarkable that IFI16 was found associated with promoters from each temporal class of HSV-1 expression (Figure 8). This suggests that the inhibition of HSV-1 by IFI16-mediated transcriptional repression is not the result of IFI16 associating with only IE promoters causing the inhibition of expression of downstream classes of genes by inhibiting their viral activators. This provides IFI16-expressing cells with redundant means of HSV-1 gene expression inhibition. However, the binding of IFI16 to each temporal class of HSV-1 gene does not exclude the possibility that it prevents other stages of HSV-1 replication. IFI16 is involved in STING-mediated type 1 IFN induction [20], [28], [67], STING is associated with the ER and trans-Golgi network [78], and HSV-1 nucleocapsids bud through the Golgi during egress [1]. IFI16 may inhibit budding of progeny HSV-1 virions. Further studies are required to investigate this possibility and also to determine the extent and localization of IFI16 binding sites to HSV-1 DNA.

We show that IFI16 prevents the association of important transcription factors with HSV-1 gene promoters (Figures 9–11). However, HSV-1 gene expression is not completely abrogated in the presence of IFI16, as shown here, and by the permissiveness of most cell types to infection with HSV-1 [1]. Therefore, the effect of IFI16 on HSV-1 gene repression cannot be absolute. This could be due to the stoichiometry of the interaction between IFI16 and HSV-1 promoters: there may not be sufficient IFI16 to promote IFN and inflammasome induction and concurrently be present on all HSV-1 promoters. The induction of IFI16 expression at early times p.i. in HFF cells [19] and U2OS cells (Figure 6) could be a cellular response to simultaneously promote all of these activities.

In addition, the HSV-1-induced degradation of IFI16 occurs with significantly slower kinetics than that of another nuclear foreign DNA sensor and viral restriction factor, PML [44]. This suggests that IFI16 has a nuanced role in the regulation of HSV-1 genes and may be useful for HSV-1 at early times p.i. Perhaps its viral gene repression activity facilitates HSV-1 replication, *in vivo*, by preventing uncontrolled viral replication and undue stress on host cells and the infection microenvironment, which may expedite immune cell recruitment. It is also possible that IFI16 may act to promote the expression of some viral genes, as it is a positive regulator of some cellular genes [48].

IFI16 is involved in cell-cycle regulation and the DNA damage response [48], [79], [80]. It could interfere with the HSV-1 DNA replication process, making it critical for the virus to decrease IFI16 protein levels prior to the bulk of DNA replication. Further studies are needed to fully appreciate the consequences of IFI16 degradation during the HSV-1 replication cycle.

It is clear that IFI16 can discriminate between host and foreign DNA [20], [28], . Some studies suggest that this is due to differences in chromatinization state [38]. Because of the global differences in chromatin modifications we observed and the activation of IFI16 during EBV and KSHV latency, during which viral DNA is chromatinized [40], [42], we believe that there must be other factors involved. Perhaps the topography of viral DNA is different from that of the host genome, leading to increased affinity, or IFI16 may bind DNA in concert with other DNA binding proteins, thereby increasing the affinity or avidity of an interaction between IFI16 and viral DNA when compared with those of IFI16 and cellular DNA.

### Potential role for IFI16 in HSV-1 latency establishment

ICP0 is a multifunctional IE alphaherpesvirus protein that is important in reactivation of HSV-1 from latency [1]. Though mechanisms of HSV-1 latency establishment are not yet well understood, ICP0 has long been considered a confounding factor. Recently a neuron-specific microRNA, miR-138, was shown to be important in the establishment of HSV-1 latency by targeting ICP0 mRNA for degradation [81]. ICP0 is necessary for the degradation of IFI16 in non-ICP0-complementing cells [19], [20], [44] and here we show that IFI16 inhibits HSV-1 gene expression. If, in neurons, miR-138 effectively disposes of ICP0, IFI16 would remain stable and able to carry out its role in gene repression. Our studies suggest that a balance between ICP0 and IFI16 may play a crucial role in determining the outcome of infection. Additionally, because the restriction of HSV-1 by IFI16 is independent of the roles IFI16 plays in the inflammasome and interferon responses (Figure 4), this offers a potential mechanism for IFI16 control of HSV-1 lytic gene expression during latency maintenance that does not lead to inflammation. IFI16 is, indeed, expressed in human neurons (Johnson, unpublished data) and further studies could elucidate its potential role in HSV-1 latency establishment.

### IFI16 and HSV-1 in U2OS cells

Like that of other innate immune factors [47], the role of IFI16 in different innate responses may be cell type-specific and modulated by HSV-1 proteins such as ICP0 and the Us3 protein kinase [44]. We showed here that IFI16 inhibits HSV-1 replication in multiple cell types (Figures 1–2), suggesting that this effect is general and not likely to be cell-type dependent.

Recent studies that point to the relative stability of IFI16 in HSV-1-infected U2OS cells [44] and more established studies suggesting that replication-defective HSV-1 ICP0 mutant viruses can successfully replicate in U2OS cells [82] caused us some concern. However, a) similar effects of IFI16 on HSV-1 replication and gene expression in HFF and U2OS cells (Figures 1 and 2), b) IFI16 degradation in HSV-1-infected U2OS cells (Figure 6) albeit at a much later time point than in HFF cells [19], [20] and, indeed, a later time point than was tested previously in U2OS cells [44], and c) enrichment of IFI16 on HSV-1 promoters in HFF cells and U2OS cells (Figure 8), demonstrate that the mechanisms of IFI16-mediated inhibition of HSV-1 gene expression are shared between cell types.

The decreased association of IFI16 with the late HSV-1 Us11 promoter in U2OS cells compared with that in HFF cells may provide insights into the increased viral yield in U2OS cells compared with that in HFF cells (Figures 1 and 2). Us11 has many diverse pro-HSV-1 functions, including promotion of protein synthesis, intracellular trafficking, inhibition of RIG-like receptor signaling, and inhibition of autophagy [83]–[88]. Further studies are essential to clarify these effects.

Importantly for the establishment of our CRISPR-mediated IFI16-negative cell line, a clonal population of U2OS cells can be grown from a single cell (Figure 5), which ensures identical genotypes for all cells tested. We were not able to grow such clonal populations of HFF cells. Given the consistency between U2OS and HFF cells described above, the U2OS and IFI16-negative clone 67 provide a valuable tool for further studies of the antiviral roles of IFI16. The slower cell growth rate and change in morphology of IFI16-negative U2OS cells could be due to the interactions of IFI16 with p53, which affect cell cycle dynamics [35]. p53 has important roles in cell morphology [89]–[92], which may also be modulated by its association with IFI16.

### Therapeutic implications

Several antiviral activities have now been described for IFI16 to counter infection by a broad range of viruses, including α- and γ-herpesviruses, HIV, and Vaccinia virus [19], [20], [28], [38]–[42], [45], [93], [94]. Developing drugs that stabilize the foreign gene repressive functions of IFI16 (this study and [19], [38], [45]) and transiently support the IFN-inducing and inflammasome-activating functions of IFI16 without allowing for constitutive innate immune signaling could lead to effective, broad range antiviral therapeutics. To safely design such a drug, future studies need to be done to further characterize the nuances of IFI16-DNA binding, IFI16-mediated IFN induction, and HSV-1-induced IFI16 degradation.

## Materials and Methods

### Cells

Human osteosarcoma cells (U2OS), human foreskin fibroblasts (HFF cells), MCF7 (breast epithelial cancer) cells, human embryonic kidney (HEK293T), and African green monkey (Vero) cells were from American Type Culture Collection (ATCC, Manassas, VA). These cells were propagated in Dulbecco's modified Eagle Medium (DMEM) supplemented with Glutamax (Gibco, Grand Island, NY), 10% fetal bovine serum (FBS; Atlanta Biologicals, Lawrenceville, GA), and 1% penicillin/streptomycin (Gibco, Grand Island, NY). They were routinely tested for mycoplasma using the Mycoalert kit (Lonza, NJ), according to the manufacturer's instructions, and were found to be negative.

### HSV-1

KOS strain HSV-1 was propagated and titered by plaque assay on Vero cells, as described [95]. Briefly, Vero cells were infected with HSV-1 at an moi of 0.001 pfu/cell until cells began to round up and could be shaken from the flask (3–5 days). At 4–6 h prior to harvest, 50 µg/mL heparin was added to cell culture supernatant. Cells were removed from cell culture supernatant by centrifugation at 1,000 rpm for 10 min at 4°C. Virus was isolated by further centrifugation of the supernatant at 20,000×g for 2 h at 4°C. Pellet was resuspended in PBS-AB/15% glycerol and stored at −80°C.

### Genome-labeled HSV-1

To generate 5-ethynyl-2′doxyuridine- (EdU) and 5-bromo-2-deoxyuridine (BrdU)-labeled infectious HSV-1 virus, a modified protocol described to produce BrdU-labeled HCMV [96] and KSHV [93] was used. Briefly, while propagating KOS strain HSV-1, EdU Labeling Reagent (Life Technologies, Camarillo, CA) was added to flasks at 50 µM and BrdU Labeling Reagent (Life Technologies) was diluted 1∶100 and added to the culture medium at 8, 24, and 48 h p.i. Flasks with media containing BrdU were kept in darkness or dim light during incubation to avoid photolysis of BrdU residues. Viral purification was carried out as described [95].

### Virus infection

Cells were incubated with HSV-1 for 2 h or until the time indicated in serum free DMEM, washed with PBS, and incubated in DMEM supplemented with 2% FBS until the times indicated.

### Plaque assays

Viral yield at 24 h p.i. was determined by titering on Vero cells. Briefly, infected cell supernatants were cleared of cell debris by centrifugation. Vero cells were infected in duplicate or triplicate with serial dilutions of supernatants for 2 h in serum free DMEM, washed with PBS, overlaid with 1× DMEM/1% agarose, and incubated at 37°C until plaque formation was observed (48–72 h). Cells were fixed by overlaying the agarose layer with 4% paraformaldehyde in PBS for 20 min and then stained with 0.2% crystal violet in 50% methanol for 20 min. Dye was washed off and plaques counted. Figures shown are representatives of three or more experiments, each.

### Generation of IFI16-negative U2OS cells

A plasmid was constructed by cloning IFI16 guided RNA (target sequence: GAAAAGTTCCGAGGTGATGCTGG synthesized within the guided RNA scaffold [54]) into pGEMT, using NheI sites. Using Lipofectamine LTX and Plus reagent (Life Technologies), U2OS cells were transfected with 3 plasmids encoding: guided RNA, Cas9 (Addgene plasmid 41815, a generous gift from Dr. George Church [54]), and GFP at a ratio of 4∶1∶1, respectively. At 48 h post-transfection, GFP-positive cells were sorted individually into 96-well plates containing complete growth media. Lack of IFI16 expression in each clone was screened by dot blot and confirmed by western blot.

### Antibodies

The following antibodies were used in Western blot and immunofluorescence analysis: mouse anti-IFI16 and anti-BrdU (Santa Cruz Biotechnology, Inc, Santa Cruz, CA), TBP (Abcam, Cambridge, MA), ASC (MBL Laboratories, Woods Hole, MA), and rabbit-anti IFI16 and anti-actin (Sigma, St. Louis, MO).

Antibodies used for chromatin immunoprecipitation assays (ChIP) were: IFI16, RNA polymerase II, TBP, Oct1, total histone H3, H3K9me3, H3K4me3, and HSV-1 VP16 (Abcam, Cambridge, MA), normal mouse IgG (Santa Cruz Biotechnology, Inc).

### Lentiviruses

To create an IFI16-expression lentiviral vector, the IFI16 coding region (NM_001206567.1, nucleotides 291–2482) was cloned into pcpsppw [97] using primers (Table 1) and the BamHI and ApaI sites. IFI16 lentiviral vectors were produced using a four-plasmid transfection system, as previously described [97]. Briefly, HEK293T cells were transfected with IFI16 expressing vector and packaging plasmids and the media was changed 16 h after transfection. Supernatants containing the lentiviral vectors were collected 24 h later, passed through a 0.44 µm filter and used to transduce cells in the presence of polybrene (5 µg/ml, Pierce, Rockford, IL). ShIFI16, shASC, and shCtrl were obtained (Santa Cruz Biotechnologies) and HFF cells were transduced according to the manufacturer's instructions. No selection was done. Western blot analysis was performed to confirm the level of knockdown at 48 h post-transduction.

**Table 1:** Primers used in this study.

Gene	Primers
**For mRNA RT-PCR - used in ** **Figures 1** **, ** **2** **, ** **3** **, and ** **6**	
**ICP0**	5′-AAGCTTGGATCCGAGCCCCGCCC-3′ (forward)
	5′-AAGCGGTGCATGCACGGGAAGGT-3′ (reverse) [100]
**ICP4**	5′-GACGTGCGCGTGGTGGTGCTGTACTCG-3′ (forward)
	5′-GCGCACGGTGTTGACCACGATGAGCC-3′ (reverse) [100]
**ICP8**	5′-GACATTACGTTCACGGCCTTCGAAGCCAG-3′ (forward)
	5′-GGCCGAGTTGGTGCTAAATACCATGGC-3′ (reverse) [100]
**TK**	5′-CGAGACAATCGCGAACATCTAC-3′ (forward)
	5′-CCCCGGCCGATATCTCA-3′ (reverse) [101]
**gB**	5′-TGTGTACATGTCCCCGTTTTACG-3′ (forward)
	5′-GCGTAGAAGCCGTCAACCT-3′ (reverse)
**Us11**	5′-CTTCAGATGGCTTCGAGATCGTAG-3′ (forward)
	5′-TGTTTACTTAAAAGGCGTGCCGT-3′ (reverse) [102]
**GAPDH**	5′-ACAGTCAGCCGCATCTTCTT-3′ (forward)
	5′-ACGACCAAATCCGTTGACTC-3′ (reverse)
**For ChIP analysis- TSS primers used in ** **figures 8** **, ** **9** **, ** **10** **, ** **11** **, and ** **12**	
**ICP0**	5′-ATAAGTTAGCCCTGGCCCCGA-3′ (forward)
	5′- GCTGCGTCTCGCTCCG-3′ (reverse) [59]
**ICP4**	5′-GCGCTCCGTGTGGACGAT-3′ (forward)
	5′-CGGCCCCTGGGACTATATGA-3′ (reverse) [13]
**ICP8**	5′-CCACGCCCACCGGCTGATGAC-3′ (forward)
	5′-TGCTTACGGTCAGGTGCTCCG-3′ (reverse) [13]
**gB**	5′-TGGGTGGAGTGATCAAAGAG-3′ (forward)
	5′- GCATCACCCATCGCTTCT-3′ (reverse)
**Us11**	5′-GTTGGGTCTGGCTCATCTC-3′ (forward)
	5′- TAATCCGGTAACCCGTTGAG-3′ (reverse)
**GAPDH**	5′- TTCGACAGTCAGCCGCATCTTCTT-3′ (forward)
	5′- CAGGCGCCCAATACGACCAAATC -3′ (reverse) [13]
**p21**	5′- GAGTCTTGCTCAGTGGGAGCTCTGGGAGTA-3′ (forward)
	5′- ATGTGACTTGGGGTGAGGCCTACTCGG-3′ (reverse)
**For IFI16 Lentiviral construction**	
**IFI16**	5′-CGCGGATCATGGAAAAAAATACAAGAA-3′ (forward)
	5′-CGCGGGCCCTTTTAGAAGAAAAAGTCTGG-3′ (reverse)

### siRNA

Scrambled siRNA and siIFI16 were transfected into HFF cells using the Neon transfection system (Life Technologies), according to the manufacturer's instructions and as described [93]. Briefly, subconfluent cells were harvested, washed once with PBS, and resuspended in resuspension buffer R (provided) at a density of 1×10^7^ cells/ml. 10 µL of this cell suspension was mixed with 100 pmol siRNA and microporated at room temperature using a single pulse of 1350 V for 30 ms. After microporation, cells were distributed into complete medium and placed at 37°C in a humidified 5% CO_2_ atmosphere. 48 h post-transfection, cells were analyzed for knockdown efficiency by western blot. siRNA oligonucleotides (siGenome SMARTpool) for IFI16 (catalog number MHSXX0020) were purchased from Thermo Fisher Scientific (Waltham, MA).

### Western blot analysis

Cells were lysed in radioimmunoprecipitation assay (RIPA) buffer (15 mM NaCl, 1 mM MgCl_2_, 1 mM MnCl_2_, 2 mM phenylmethylsulfonyl fluoride) supplemented with protease inhibitor cocktail (Sigma), sonicated, and clarified by centrifugation at 16,000×g for 10 min. Equal amounts of protein were separated by SDS-PAGE and electrophoretically transferred to nitrocellulose membranes. Membranes were incubated with primary antibodies and secondary antibodies conjugated to horseradish peroxidase (KPL, Gaithersburg, MD). Immunoreactive bands were visualized using ECL western blotting substrate (Pierce). Blots were scanned using FluorChemFC2 software with the AlphaImager system (Alpha Innotech Corporation, San Leonardo, CA). Figures shown are representatives of three or more experiments each.

### Immunofluorescence analysis

Cells in 8-chamber slides were infected as indicated before fixation for 10 min with 4% paraformaldehyde. They were washed with PBS, permeabilized with 0.2% Triton X-100 for 10 min, and blocked with Image-IT FX signal enhancer (Life Technologies) for 10 min. Cells were incubated with primary antibodies for 1 hour at room temperature in PBS, 1% BSA, washed, and incubated with Alexa fluor-conjugated secondary antibodies for 1 hour at room temperature in PBS, 1% BSA. They were washed again and mounted onto slides using Slowfade gold mounting reagent with DAPI (Life Technologies). BrdU-labeled virus was detected as described [93]. Briefly, cells were fixed for 10 min with 4% paraformaldehyde then treated with 4N HCl for 10 min at room temperature to expose BrdU to antibody staining. Cells were washed with PBS, permeablized with 0.2% Triton X-100 for 10 min, and blocked with Image-IT FX signal enhancer (Life Technologies) for 10 min. Cells were stained with a rabbit-derived antibody to BrdU and Alexa Fluor-conjugated secondary antibodies (Life Technologies) and mounted onto slides as above. Cells were imaged using a Nikon Eclipse 80i fluorescence microscope and Metamorph software (Molecular Devices, Silicon Valley, CA). Figures shown are representatives of two or more experiments each.

### RNA purification and cDNA synthesis

Total RNA was extracted from cells using Trizol (Life Technologies), according to the manufacturer's instructions. Briefly, cells were homogenized in Trizol and mixed with chloroform to separate the proteins and nucleic acids. RNA was precipitated from the aqueous layer using isopropanol and washed in 75% ethanol before resuspension in RNase-free water. RNA was DNase treated for 30 min at 37°C (Promega, Madison, WI) and reverse transcribed using Multiscribe Reverse Transcriptase (Life Technologies) with random primers, according to the manufacturer's instructions.

### Chromatin immunoprecipitation

To perform ChIP assays, we used a protocol modified from two previous studies [98], [99]. Briefly, 90–95% confluent T-150 flasks of cells were cross-linked for 10 min by adding formaldehyde to a final concentration of 1%. Crosslinking was stopped by adding glycine to 125 mM for 5 min. Cells were collected and resuspended in cell lysis buffer (5 mM PIPES, pH 8.0; 1 mM EDTA; 1% SDS; protease inhibitors) and incubated on ice for 15 min before being passed through a 27.5 gauge needle and a 30 gauge needle 5 times, each. Nuclei were pelleted by centrifugation and then resuspended in nuclear lysis buffer (50 mM Tris, pH 8.1; 10 mM EDTA; 1% SDS; protease inhibitors), incubated on ice for 15 min, and sonicated at an amplitude of 40, 10 seconds on, 10 seconds off for 20 min. Debris was cleared by centrifugation and supernatant was flash frozen in liquid N_2_ and stored at −80°C overnight. Nuclear lysates were diluted in ChIP dilution buffer (0.01% SDS; 1.1% Triton X-100; 1.2 mM EDTA; 16.7 mM Tris, pH 8.1; 167 mM NaCl; protease inhibitors). Diluted lysates were precleared for 30 min at 4°C with salmon sperm DNA/protein G agarose slurry (Millipore, Billerica, MA) and then incubated overnight at 4°C with 1.5 µg of the indicated antibody. Immune complexes were collected with salmon sperm DNA/protein G agarose slurry for 1 hour at 4°C and washed with low salt wash (0.1% SDS; 1% Triton X-100; 2 mM EDTA; 20 mM Tris, pH 8.1; 150 mM NaCl), then high salt wash (0.1% SDS; 1% Triton X-100; 2 mM EDTA; 20 mM Tris, pH 8.1; 500 mM NaCl), and LiCl wash (0.25 M LiCl; 1% NP-40; 1% deoxycholate; 1 mM EDTA; 10 mM Tris, pH 8.1). Complexes were eluted in elution buffer (1% SDS; 0.1 M NaHCO_3_). The crosslinking was reversed by adding 1 µL RNase A and NaCl to a final concentration of 0.3 M NaCl and incubating at 65°C for 5 hours. Protein was removed by incubating lysate with proteinase K at 55°C for 1 hour. DNA was precipitated with ethanol and resuspended in nuclease-free water before real-time PCR with TSS primers (Table 1).

### Real-time PCR

Real-time PCR was performed using SYBR green (Life Technologies) and primers (Table 1) according to the manufacturer's instructions. Briefly, 2 µL of cDNA (for mRNA experiments) or immunoprecipitated DNA (for ChIP experiments) was added to real-time PCR reaction mixtures containing SYBR green reaction mixture (final concentration of 1×) and the appropriate primers (final concentration of 0.25 µM, forward and reverse). An ABI Prism 7500 real-time PCR system was used to amplify and detect cDNA. cDNA data were normalized to GAPDH Ct levels and ChIP data were normalized to input samples, as indicated, using the ddCt method [46]. Figures shown are representatives of three or more experiments each.

### In situ proximity ligation assay (PLA) microscopy

PLA was performed using the DuoLink PLA Kit (Sigma-Aldrich) to detect protein–protein interactions using fluorescence microscopy as per manufacturer's protocol. Briefly, HFF cells were cultured and infected with EdU-labeled HSV-1 (1 pfu/cell) for the indicated times in 8 chamber microscopic slides, fixed with 4% paraformaldehyde for 15 minutes at room temperature, permeabilized with 0.2% Triton X-100 and blocked with DuoLink blocking buffer for 30 minutes at 37°C. Cells were then incubated with primary antibodies against IFI16 (mouse monoclonal and rabbit polyclonal), diluted in DuoLink antibody diluents for 1 hour, washed and then further incubated for an hour at 37°C with species-specific PLA probes under hybridization conditions and in the presence of 2 additional oligonucleotides to facilitate the hybridization only in close proximity (<40 nm). Hybridized oligonucleotides were ligated to form a closed circle, which served as a template for rolling-circle amplification after adding an amplification solution to generate a concatemeric product extending from the oligonucleotide arm of the PLA probe. Fluorescently labeled oligonucleotides were hybridized to the concatemeric products and the signal was detected as distinct fluorescent dots in the Texas red channel and analyzed by fluorescence microscopy as above.

### Interferon-β ELISA

IFNβ secretion was detected using the *Verikine™* Human IFN Beta ELISA kit (PBL Interferon Source, Piskataway, NJ) according to the manufacturer's instructions. Infected cell supernatant was collected at 6 h p.i., diluted 1∶1 with sample dilution buffer and attached to the assay wells by incubation at room temperature for 1 h. Wells were washed and incubated 1 h at room temperature with diluted antibody solution, then washed again and incubated 1 h at room temperature with diluted HRP solution. Wells were washed again and incubated 15 min at room temperature with TMB substrate solution in the dark. The reactions were stopped by the addition of stop solution and the absorbance at 450 nm was read using a Synergy2 Biotek plate reader (Biotek, Winooski, VT).

## Supporting Information

Figure S1 **Immunofluorescence analysis of BrdU labeled HSV-1 genome entry into the nuclei of HFF, U2OS, and IFI16-negative U2OS cells.** HFF (A), U2OS (B), and clone 67 (C) cells were mock infected or infected with BrdU-labeled HSV-1 (moi of 1 pfu/cell) for 15, 30, and 90 minutes. Immunofluorescence of BrdU (green) is shown and the nuclei were counterstained with DAPI.
(TIF)
