# Correction: IFI16 Restricts HSV-1 Replication by Accumulating on the HSV-1 Genome, Repressing HSV-1 Gene Expression, and Directly or Indirectly Modulating Histone Modifications

**DOI:** 10.1371/journal.ppat.1007113

**Published:** 2018-06-06

**Authors:** Karen E. Johnson, Virginie Bottero, Stephanie Flaherty, Sujoy Dutta, Vivek Vikram Singh, Bala Chandran

The authors would like to correct two errors in Fig 8. The figure contains two errors. In Fig 8F, gel panels gB and Us11 are duplicate. In Fig 8G, gel panels HFF and U2OS are duplicates. These errors occurred during the resubmission of the manuscript.

The authors have informed the publisher that they are not able to provide the original raw data for this figure because the lab closed January 2017.

However, the authors confirm that the duplicate panels in Fig 8 contained minor supportive data of their ChIP data showing IFI16 binds to the various HSV-1 promoters in IFI16 positive U2OS cells and not in U2OS IFII16 knockout cells (U2OS clone 67) cells.

The authors have provided a corrected Fig 8 and Fig 8 legend here. The authors confirm that these changes do not alter their findings.

**Fig 8:**
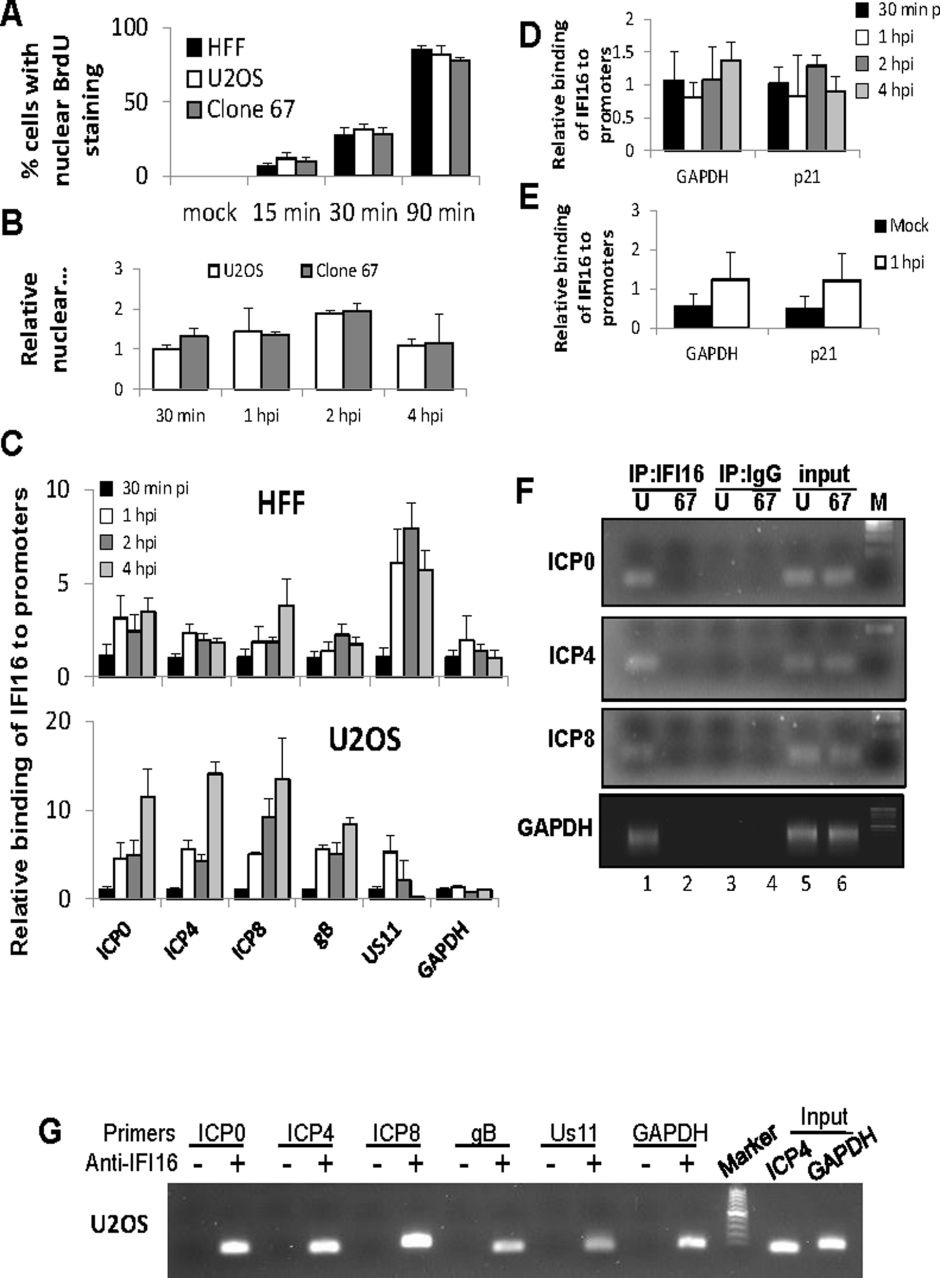
ChIP analysis of IFI16 binding to the HSV-1 genome at the transcriptional start site of viral genes. (A) HFF, U2OS, and clone 67 cells were infected with BrdU-labeled HSV-1 at an moi of 1 pfu/cell for 15, 30, or 90 min before fixation. Cells were immunostained for BrdU. The percent of cells with nuclear BrdU was quantified by scoring BrdU localization in 4 fields of cells per sample (total 150–200 cells/sample). (B–G) U2OS and clone 67 cells were mock infected or infected with wt HSV-1 at an moi of 1 pfu/cell. (B) The relative quantity of nuclear HSV-1 DNA at various times post infection was quantified by qPCR of HSV-1-infected nuclear extracts from U2OS and clone 67 U2OS cells using primers for the ICP4 start site. (C) ChIP analysis of IFI16 in HFF and U2OS cells. IFI16 was immunoprecipitated from cells infected with HSV-1 (1 pfu/cell) for 30 min, 1, 2, or 4 hours. Bound DNA was analyzed by real-time PCR with primers to regions flanking the transcriptional start sites of the genes indicated. Values were normalized to IFI16-bound GAPDH and input ICP4. (D) ChIP analysis of IFI16 binding the cellular promoters for GAPDH and p21 in U2OS cells, performed as above. (E) ChIP analysis of IFI16 binding cellular promoters in uninfected U2OS cells or cells infected with HSV-1 at an moi of 1 pfu/cell for 1 hour, performed as above. (F) Agarose gels showing HSV-1 and cellular promoter DNA precipitated with antibody to IFI16 or control IgG in U2OS (U) or clone 67 (67) cells at 1 h p.i., and an moi of 1 pfu/cell. (G) Agarose gels showing the amplification of the indicated HSV-1 and cellular promoter regions after IFI16 ChIP at 4 h p.i.

## References

[1] Johnson KE, Bottero V, Flaherty S, Dutta S, Singh VV, Chandran B (2014) IFI16 Restricts HSV-1 Replication by Accumulating on the HSV-1 Genome, Repressing HSV-1 Gene Expression, and Directly or Indirectly Modulating Histone Modifications. PLoS Pathog 10(11): e1004503 https://doi.org/10.1371/journal.ppat.1004503 25375629 10.1371/journal.ppat.1004503 PMC4223080

