# Supplementary figures and images for: IFI16 Restricts HSV-1 Replication by Accumulating on the HSV-1 Genome, Repressing HSV-1 Gene Expression, and Directly or Indirectly Modulating Histone Modifications
Source: PLoS Pathog. 2014 Nov 6;10(11):e1004503. doi: 10.1371/journal.ppat.1004503 (PMC4223080; doi:10.1371/journal.ppat.1004503)

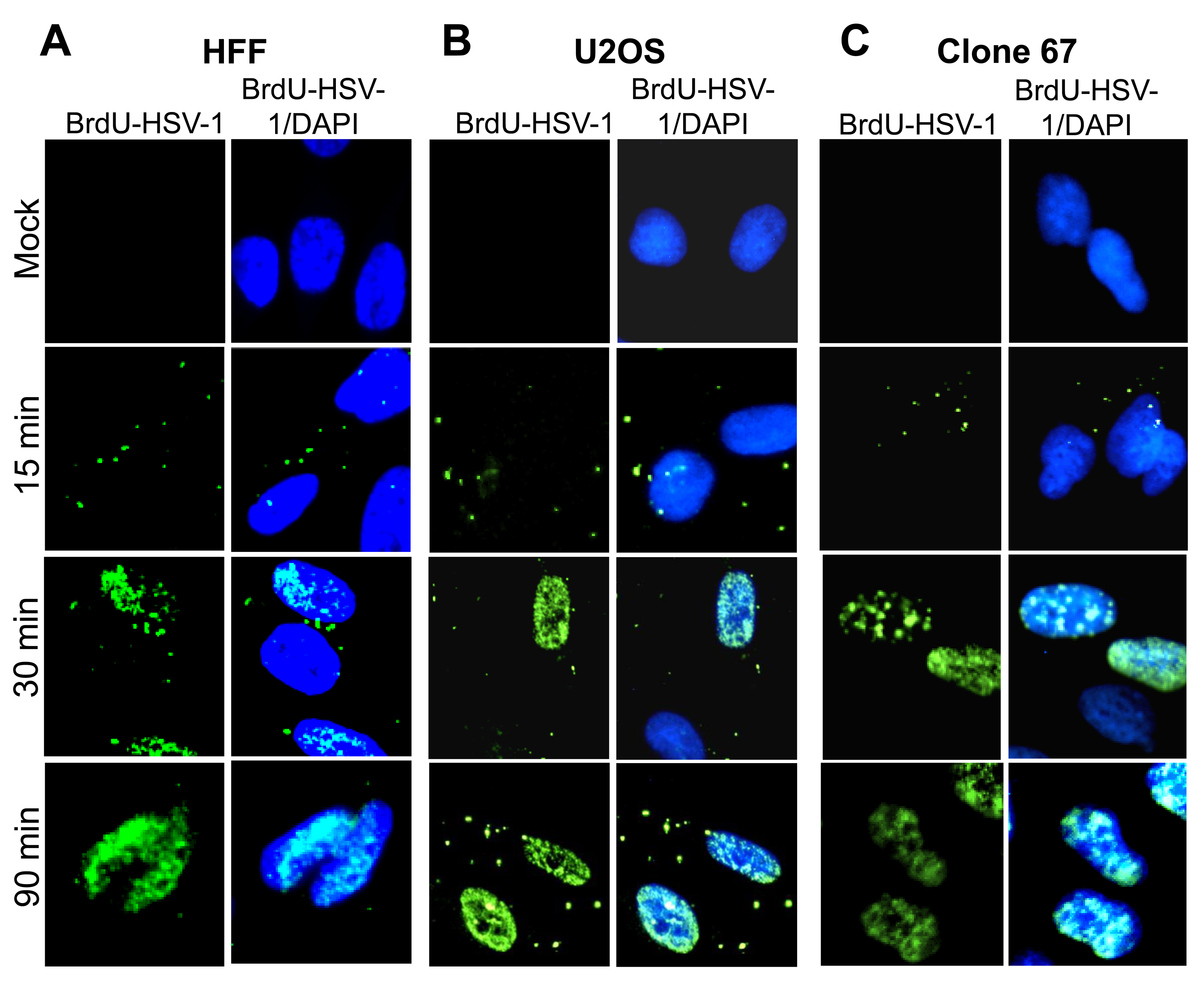

Supplement: Figure S1 — Immunofluorescence analysis of BrdU labeled HSV-1 genome entry into the nuclei of HFF, U2OS, and IFI16-negative U2OS cells. HFF (A), U2OS (B), and clone 67 (C) cells were mock infected or infected with BrdU-labeled HSV-1 (moi of 1 pfu/cell) for 15, 30, and 90 minutes. Immunofluorescence of BrdU (green) is shown and the nuclei were counterstained with DAPI. (TIF) [file ppat.1004503.s001.tif]
